# Time‐specific convergence and divergence in individual differences in behavior: Theory, protocols and analyzes

**DOI:** 10.1002/ece3.10615

**Published:** 2023-11-28

**Authors:** Judy A. Stamps, Peter A. Biro

**Affiliations:** ^1^ Department of Evolution and Ecology University of California, Davis Davis California USA; ^2^ School of Life and Environmental Sciences Deakin University Geelong Victoria Australia

**Keywords:** emergence of individual differences, emergence of personality, fanning in, fanning out, repeatability, residual variance, temporal reaction norms

## Abstract

Over the years, theoreticians and empiricists working in a wide range of disciplines, including physiology, ethology, psychology, and behavioral ecology, have suggested a variety of reasons why individual differences in behavior might change over time, such that different individuals become more similar (convergence) or less similar (divergence) to one another. Virtually none of these investigators have suggested that convergence or divergence will continue forever, instead proposing that these patterns will be restricted to particular periods over the course of a longer study. However, to date, few empiricists have documented time‐specific convergence or divergence, in part because the experimental designs and statistical methods suitable for describing these patterns are not widely known. Here, we begin by reviewing an array of influential hypotheses that predict convergence or divergence in individual differences over timescales ranging from minutes to years, and that suggest how and why such patterns are likely to change over time (e.g., divergence followed by maintenance). Then, we describe experimental designs and statistical methods that can be used to determine if (and *when*) individual differences converged, diverged, or were maintained at the same level at specific periods during a longitudinal study. Finally, we describe why the concepts described herein help explain the discrepancy between what theoreticians and empiricists mean when they describe the “emergence” of individual differences or personality, how they might be used to study situations in which convergence and divergence patterns alternate over time, and how they might be used to study time‐specific changes in other attributes of behavior, including individual differences in intraindividual variability (predictability), or genotypic differences in behavior.

## Graphical abstract

Many investigators from a wide array of disciplines have predicted that individuals will become more similar to one another (convergence) or less similar to one another (divergence) during specific periods over the course of a longer study. We first consider situations in which we might observe time‐specific changes in individual differences, and then describe experimental protocols and statistical models that empiricists can use to determine if (and when) individual differences in behavior converge or diverge during a longitudinal study.

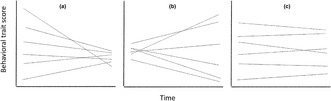

## 1 INTRODUCTION

**FIGURE 1:**
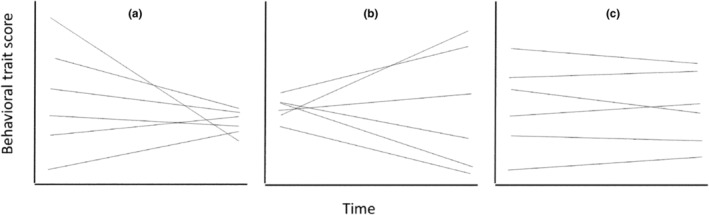
Illustration of three ways that individual differences in expected (mean) levels of behavior might change over a specific period of time. Depicted are the temporal reaction norms (temporal trendlines) of six individuals. Panel (a) illustrates convergence, a pattern in which individual differences in expected values decrease over the period. Panel (b) illustrates divergence, a pattern in which individual differences in expected values increase over the period. Panel (c) illustrates maintenance, a pattern in which individual differences in expected values are largely maintained over the period.

Empiricists studying animal personality, coping styles, and behavioral syndromes have documented hundreds of cases in which individuals differ in the levels of behavior that they express (Bell et al., 2009; Carter et al., 2013; Dougherty & Guillette, 2018; Franklin et al., 2022), and similar patterns have been described for physiological traits (Fanson & Biro, 2018; Nespolo & Franco, 2007; Taff et al., 2018; White et al., 2013). Now, theoreticians and empiricists are beginning to ask second‐order questions about individual differences in behavior. One such question is whether individual differences might change as a function of time (or age, number of trials, etc.), and if so, when those changes might occur. Although individual behavior can differ in many respects (e.g., see the discussion of individual differences in intraindividual variability at the end of this article), to date most empirical and theoretical studies have focused on individual differences in expected (mean) levels of behavior. Hence, we also focus on such differences in this article. Changes over time in the expected values of an individual are indicated by its “temporal reaction norm”. When different individuals are monitored over a particular period of time, their temporal reaction norms might converge toward one another (a “fanning in” pattern, Figure 1a), diverge from one another (a “fanning out” pattern, Figure 1b), or be maintained over that period (“maintenance,” Figure 1c).

**FIGURE 2:**
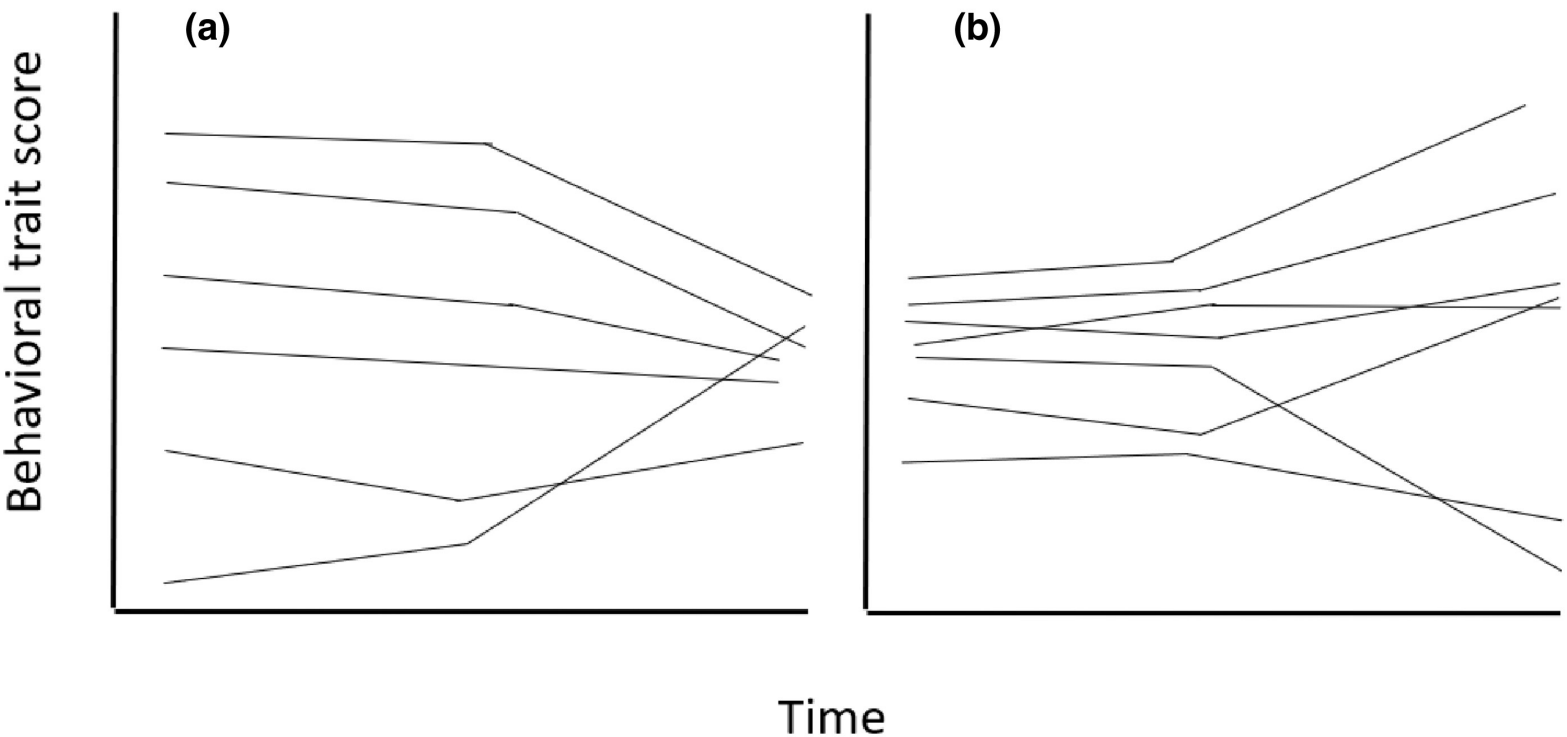
Two possible ways in which patterns of temporal change in individual differences in expected (mean) levels of behavior might change over time. Depicted are the temporal reaction norms (temporal trendlines) of six individuals, showing (a) a period of maintenance followed by a period of convergence, and (b) a period of maintenance followed by a period of divergence.

More important, as we describe in the following section, a wide range of theoretical and empirical studies suggest that if convergence or divergence patterns do occur, they will be restricted to specific periods of time. This is true of situations in which temporal changes in behavior are largely attributed to the subjects' exposure to external stimuli (as in habituation), and those in which temporal changes in behavior are largely attributed to changes in the subjects' internal state (as in changes in behavior around the time of puberty). We illustrate two simple scenarios of such patterns in Figure 2.

As a result, empiricists interested in studying temporal changes in individual differences should consider experimental designs and statistical methods which allow them to determine when, over the course of a longer study, those changes occurred. At present, however, many of those individuals are unfamiliar with the experimental designs and statistical methods that are appropriate for this task. Hence, in this article, we not only outline many of the hypotheses that predict time‐specific convergence and divergence, but also describe and provide detailed worked examples of experimental protocols and statistical methods that empiricists might use to describe those patterns. These worked examples are used to introduce readers to experimental methods and analytical approaches borrowed from quantitative genetics and other disciplines that can be used to analyze temporal changes in individual differences. Our goal is to show how fitting different models to a dataset can provide insights into the temporal patterns in that data; our aim was not to evaluate the *performance* of different models in capturing temporal trends in datasets, as that would require extensive simulations which are beyond the scope of this article (see also Section 5). The ability to determine if and when temporal changes in individual differences occur is not only required to test existing hypotheses about the evolution and adaptive significance of time‐specific convergence or divergence in individual differences, but it is also a prerequisite for studies of the proximate processes and mechanisms that might be responsible for generating them.

## 2 WHY AND WHEN WE MIGHT EXPECT TO OBSERVE TIME‐SPECIFIC CONVERGENCE OR DIVERGENCE

Convergence and divergence patterns can be described over multiple timescales, ranging from minutes to hours (e.g., in studies of habituation or sensitization) to a lifetime (e.g., in studies of the development of personality). To date, behavioral ecologists interested in convergence and divergence patterns have mostly focused on changes in individual differences across developmental or ontogenetic timescales. The extensive recent literature on animal personality is, at least in part, responsible for some of this attention. Indeed, phrases such as “the development of personality,” “the emergence of individual differences,” or “the emergence of personality” implicitly assume that divergence occurs during a specific period during ontogeny. Thus, Sih et al. (2015) devoted a review to “understanding the emergence of personality differences,” and focused on theoretical models which predict “fanning out” patterns for behavioral traits (see below). Other theoretical models predict convergence over ontogenetic timescales, for example, situations in which substantial individual differences in behavior exist at birth, hatching, eclosion, sexual maturity, or other important life‐history landmarks, but decline later in life (Stamps & Krishnan, 2014a). Convergence and divergence patterns over ontogenetic timescales were briefly reviewed in Stamps and Biro (2016). Since then, a few empiricists have looked for convergence or divergence patterns in longitudinal studies of behavioral development (e.g., Class & Brommer, 2016; Laskowski et al., 2022; Sakai, 2020, see also below).

While interest in temporal convergence and divergence patterns is relatively new to behavioral ecology, this topic has long attracted the attention of scientists interested in individual differences in the physiology and behavior of humans. Nearly 100 years ago, Wilder (1931) proposed the “Law of Initial Values,” primarily based on descriptions of convergence in human psychophysical responses (e.g., changes in heart rate or skin conductance) in response to various stimuli. The Law of Initial Values predicts negative covariance, across subjects, between their initial scores and the extent and direction of changes in their scores in response to repeated exposure to the same stimuli. The Law of Initial Values provided impetus for many empirical studies in the first half of the last century (see review in Wilder, 1965), but it fell out of favor as a result of statistical issues, in particular, with problems related to “regression to the mean” (see below). Convergence and divergence patterns have also attracted attention from psychologists interested in how humans learn skills. Thus, Ackerman (2007) considered “two enduring issues associated with skill acquisition: whether individuals become more alike in performance or more different over the course of skill acquisition.”

However, demonstrating that individual differences converge or diverge as a function of time or of the number or rate of previous experiences is just the first step in describing temporal changes in individual differences. This is because virtually no one assumes that convergence or divergence will continue “forever.” Instead, empiricists and theoreticians alike are interested in identifying periods in which convergence, divergence, or maintenance might occur. For instance, models of personality development in animals assume that convergence or divergence in expected trait values occurs early in ontogeny, and that maintenance occurs later in ontogeny. Similarly, empirical studies of learning have shown that if individuals who begin with very different scores are subjected to the same training regime, their scores typically become more similar to one another, but that modest individual differences in scores may be indefinitely sustained even after extensive training. Indeed, there may even be situations in which convergence and divergence patterns alternate with one another over time, for example, when individuals who have similar expected values at one time of year have very different expected values at another time of year (see Section 5).

### 2.1 Conditions favoring convergence

On any timescale, convergence occurs when individual differences in the expected values of a behavioral or physiological trait exist at the beginning of a period, and these differences are reduced by the end of that period. Convergence of expected values is predicted by many learning models (e.g., Rescorla & Wagner, 1972; Tarantola et al., 2017; Trimmer et al., 2012), and empiricists have demonstrated convergence patterns for many types of learning. In such cases, convergence ceases when the subjects approach asymptotic scores for the type of learning in question. For instance, researchers studying habituation often report substantial differences among their subjects in their responses the first time they are exposed to the stimulus, but find that their responses converge on more similar scores after repeated exposure to the stimulus (review in Ogorman, 1977, see also Avery & Blackford, 2016; Cavanagh et al., 2018; Colombo & Mitchell, 2009). Empiricists studying other types of learning also frequently report that individuals express different initial scores at the onset of the study, but that their scores later converge as a result of repeated exposure to the same training regime (e.g., Anglim & Wynton, 2015; Fatima et al., 2016; Langley et al., 2018; Tarantola et al., 2017). Convergence patterns are also regularly observed when humans learn simple repetitive motor skills in which performance is measured by speed and accuracy (review in Ackerman, 2007). Learning from conspecifics can also promote convergence in behavior, as has been described for vocal signals in bats (Knοrnschild et al., 2012) and foraging behavior in birds (Franks et al., 2020).

Over ontogenetic timescales, Bayesian models of development predict convergence in certain circumstances. These models consider situations in which the behavior expressed by an individual is directly related to its information‐state (e.g., antipredator behavior expressed in response to estimates of predator density), when individuals initially differ with respect to their information‐states, and when all of the subjects are repeatedly or continuously exposed to cues which provide them with the same information about the external environment (Fawcett & Frankenhuis, 2015; Stamps & Frankenhuis, 2016; Stamps & Krishnan, 2014a). In such cases, if every subject is reared in the presence of the same moderately reliable cues, these models predict convergence in the subjects' behavior over time. Moreover, they predict that the rate of convergence will gradually decline over ontogeny, such that following a period of convergence, different individuals may either express the same levels of behavior (no individual differences, e.g., see Fawcett & Frankenhuis, 2015) or they may consistently express different levels of behavior (maintenance, e.g., Stamps & Krishnan, 2014a, 2014b). Other models based on feedback loops between behavioral and state variables also predict convergence during specific periods of time (Sih et al., 2015). However, since most of these models have focused on divergence patterns, we defer discussion of them to the next section.

### 2.2 Conditions favoring divergence

On any timescale, divergence occurs when individuals who express similar expected values of behavioral or physiological traits at the beginning of a period express different expected values at the end of that period. Over 100 years of carefully controlled experimental studies of learning and other forms of developmental plasticity have shown that initially similar subjects often develop different phenotypes if they are continuously or repeatedly exposed to different stimuli or experiences (reviewed by Pigliucci, 2001; Shettleworth, 2010; West‐Eberhard, 2003). However, such studies typically do not report that divergence continues forever; instead, it usually declines and eventually ceases when the subjects reach a particular age or stage of life.

There are at least two possible reasons why free‐living animals born at the same time and locality might be consistently exposed to different environment conditions over the course of development. First, individuals might differ in their preferences for particular types of microhabitats, social situations, food items, or other features in the local environment (“niche‐picking,” or “selection of the environment”), and second, individuals might consistently differ with respect to traits that affect the social or physical environments in which they will subsequently develop (i.e., “niche construction” or “adjustment of the environment,” see Edelaar & Bolnick, 2019; Fokkema et al., 2021; Plomin et al., 1977; Scarr & McCartney, 1983; Trappes et al., 2022). In turn, if individual differences in preferences or behavior increase the probability that different individuals will be consistently exposed to different environmental conditions during specific periods during ontogeny, and if consistent exposure to different environmental conditions during those periods encourages the development of different phenotypes, one would expect to observe the divergence in phenotypes during those periods. For instance, experimental studies of red knots (*Calidris canutus islandica*) suggest that individual differences in dietary preferences may be responsible for the development of individual differences in both gizzard size and foraging behavior (patch resident times; Oudman et al., 2016). Historically, much of the literature on niche‐picking and niche‐construction has focused on situations in which initial differences in preferences or behavior have a genetic basis, leading to correlations between genotypes and the environments in which those genotypes will develop (Fokkema et al., 2021; Plomin et al., 1977, 2016; Saltz & Nuzhdin, 2014; Scarr & McCartney, 1983). However, it is clear that prior experiences, parental effects, differences in internal state, and other nongenetic factors could also encourage initial differences among individuals in preferences or trait values which would, in turn, contribute to differences among them in experiences affecting their subsequent development (Davis & Stamps, 2004; Edelaar & Bolnick, 2019; Perkeybile & Bales, 2017; Ventura & Worobey, 2013; Wilson & McLaughlin, 2007).

One often‐overlooked type of niche‐construction occurs when individuals in the same population vary with respect to traits that evoke different types of social behavior from conspecifics (Moore et al., 1997; Plomin et al., 1977; Stamps & Groothuis, 2010; Stamps & Luttbeg, 2022). For instance, in mosquitofish (*Gambusia holbrooki*), a focal male's color affects the social behavior it elicits from other adults. When males were first introduced to established social groups, silver males were chased more frequently by the resident males and followed nonaggressively more by the resident females than were melanic males (Horth, 2003). In turn, if different phenotypes in focal individuals elicit different social behaviors from conspecifics, one would expect divergence over time in the focal individuals in any trait whose development was affected by those behaviors. Thus, it is suspected that at least some of the differences in the social behavior expressed by melanic and silver males in both the laboratory and the field might be due to consistent differences in the social behavior that each of those morphs elicited earlier in life from conspecifics (Kraft et al., 2016, 2018).

The literature on social niche specialization posits that divergence in behavior over the course of ontogeny occurs as a result of niche‐construction, niche‐picking, or both (Bergmuller & Taborsky, 2010; Montiglio et al., 2013). That is, initially similar individuals might gradually adopt different behaviors as a result of receiving different behaviors from conspecifics, as a result of their preferentially adopting different social roles, or some combination of these. Again, it is assumed that divergence in behavior as a result of social niche specialization would not continue indefinitely, but that it would be followed by a period in which different individuals consistently expressed different behaviors (i.e., maintenance).

Divergence patterns for mean trait values can also occur even if initially similar individuals are all exposed to the same experiences or environmental conditions. For instance, divergence patterns for learning and cognitive skills have been described when individuals who begin with similar initial scores approach different asymptotic scores in response to the same training regime (e.g., Burki et al., 2014; Rast & Zimprich, 2009). Ackerman (2007) suggested that with respect to skill development, divergence patterns are most likely for complex skills in which performance depends heavily on domain‐specific knowledge, attentiveness, and use of working memory. In other words, even if different subjects were all exposed to an identical training regime, differences among them in a variety of traits which affect their performance might encourage divergence in their scores over time. Thus, longitudinal studies of advanced chess players have shown that for the same amount of practice (number of games played), ranking scores indicative of performance in tournaments diverged across the players across a period of decades (Howard, 2009). More broadly, scores for reading, mathematical, and other complex skills in humans often diverge as a function of age in children (Geary et al., 2009; Lohman, 1999; Stanovich, 1986), although in such cases uncontrolled experiences outside of the classroom (e.g., the amount of recreational reading) might also differ among the subjects.

Over ontogenetic timescales, Bayesian models of development predict divergence patterns under certain circumstances, even if every subject is repeatedly exposed to the same moderately reliable cues. In particular, if different individuals begin with similar estimates of conditions in the external environment (e.g., they begin with similar estimates of mean predator density) but differ with respect to their uncertainty about the accuracy of those initial estimates (indicated by the variance of the individual's initial prior, Stamps & Frankenhuis, 2016), these models predict divergence patterns for both their estimates of predator density and any behaviors related to those estimates. That is, these models predict that plasticity in response to the same experience will differ among individuals, depending on differences among them in the variance of their prior distributions at the onset of that experience. In such cases, these models predict that a period of strong divergence early in ontogeny will be followed by a period approximating maintenance later in ontogeny (Stamps & Krishnan, 2014a, 2014b).

Fisher et al. (2018) recently suggested that divergence patterns for mean values over ontogenetic timescales might occur as a result of chaotic dynamics. They argued that even minor variation across individuals early in development could, as a result of nonlinear, multiplicative interactions during development, encourage a gradual divergence in mean values for behavior later in life. This hypothesis was suggested by reports indicating that individual differences in behavior are observed even after iso‐genetic subjects have been reared in virtually identical social and physical environments (e.g., Bierbach et al., 2017; Polverino et al., 2016). However, although chaotic dynamics might account for divergence in the behavior of initially nearly identical subjects, Fisher et al. note that one must add assumptions to their model (e.g., that chaotic dynamics only occur early in life) to explain why divergence would not continue indefinitely, but instead decline later in ontogeny.

Sih et al. (2015) reviewed a range of models in behavioral ecology which suggest that feedbacks between behavioral and state variables might encourage either convergence or divergence patterns in the mean values of both. Because these authors were primarily interested in the “emergence of personality,” they focused on models which demonstrate that positive feedbacks between a state variable and a behavior can encourage divergence patterns for both the state variable and the behavior, where “state variable” was very broadly defined as “any feature that affects the cost or benefits of the behavioral action.” For example, if individuals in good condition behave more boldly when foraging, and if higher foraging rates enhance body condition, one would expect divergence across individuals in both boldness in a foraging context and condition (Luttbeg & Sih, 2010). Verbal models suggesting that positive feedback loops between behavior and state might contribute to the development of personality have also appeared in the psychology literature. For instance, the “corresponsive principle of personality development” (Caspi et al., 2005) posits that individuals with particular personality traits initially seek out particular social situations, and that spending time in those social situations deepens and enhances the personality traits that led those individuals to seek them out in the first place.

In contrast with other explanations for divergence (see above), positive feedback models predict that both the behavior of interest and the state variable that affects the fitness consequences of that behavior will change over time, and that the behavioral variable and the state variable will be correlated with one another over time within individuals. In principle, minor, even stochastic, differences among individuals early in life in either the state variable or the behavior could “get the ball rolling.” However, in the absence of additional assumptions, these models predict that divergence due to positive feedback would continue indefinitely. Sih et al. (2015) readily acknowledge this problem, suggesting that “individual divergence due to positive feedback would typically cease at some point in time either because of biological floors or ceilings to both state and behavior, because behavior is open for modification only during certain developmental stages, or because the effect of state on behavior (or vice versa) is non‐linear.”

Sih et al. (2015) also reviewed several models which show how negative feedbacks between state variables and behavior might lead to convergence patterns. Indeed, many of the same models predict either positive feedback (and divergence) or negative feedback (and convergence), depending on assumptions about other variables. For instance, models of relationships between energy reserves and food sampling behavior can predict either convergence or divergence patterns, depending on assumptions about the risk of starvation in the local environment (Mathot & Dall, 2013). Such models imply that convergence and divergence might alternate over time within the same set of individuals, for example, if seasons with food abundance alternated with seasons of food scarcity. As was the case for divergence, negative feedback models which predict convergence indicate that both the behavior and the state variable will change over time, and that the behavior and the state variable will be correlated within individuals over time.

## 3 HOW CAN EMPIRICISTS DETERMINE IF (AND WHEN) INDIVIDUAL DIFFERENCES CHANGE OVER TIME?

### 3.1 Practical concerns

In order to characterize temporal changes in individual differences in behavior, we need to measure the same behavior in the same subjects at different periods over the course of a study. That is, these analyzes require a longitudinal rather than a cross‐sectional, experimental design. In addition, the patterns illustrated in Figures 1 and 2 are based on the expected values of each subject at different points in time, not on statistics based on their cumulative scores over time. For instance, divergence patterns for space use behavior have been described for genetically identical mice housed in large, complex arenas (Freund et al., 2013, 2015; Torquet et al., 2018). However, since the estimates of space use in these studies were based on a cumulative measure (roaming entropy), the extent to which the behavior of the subjects actually diverged over the course of the study is unclear.

**FIGURE 3:**
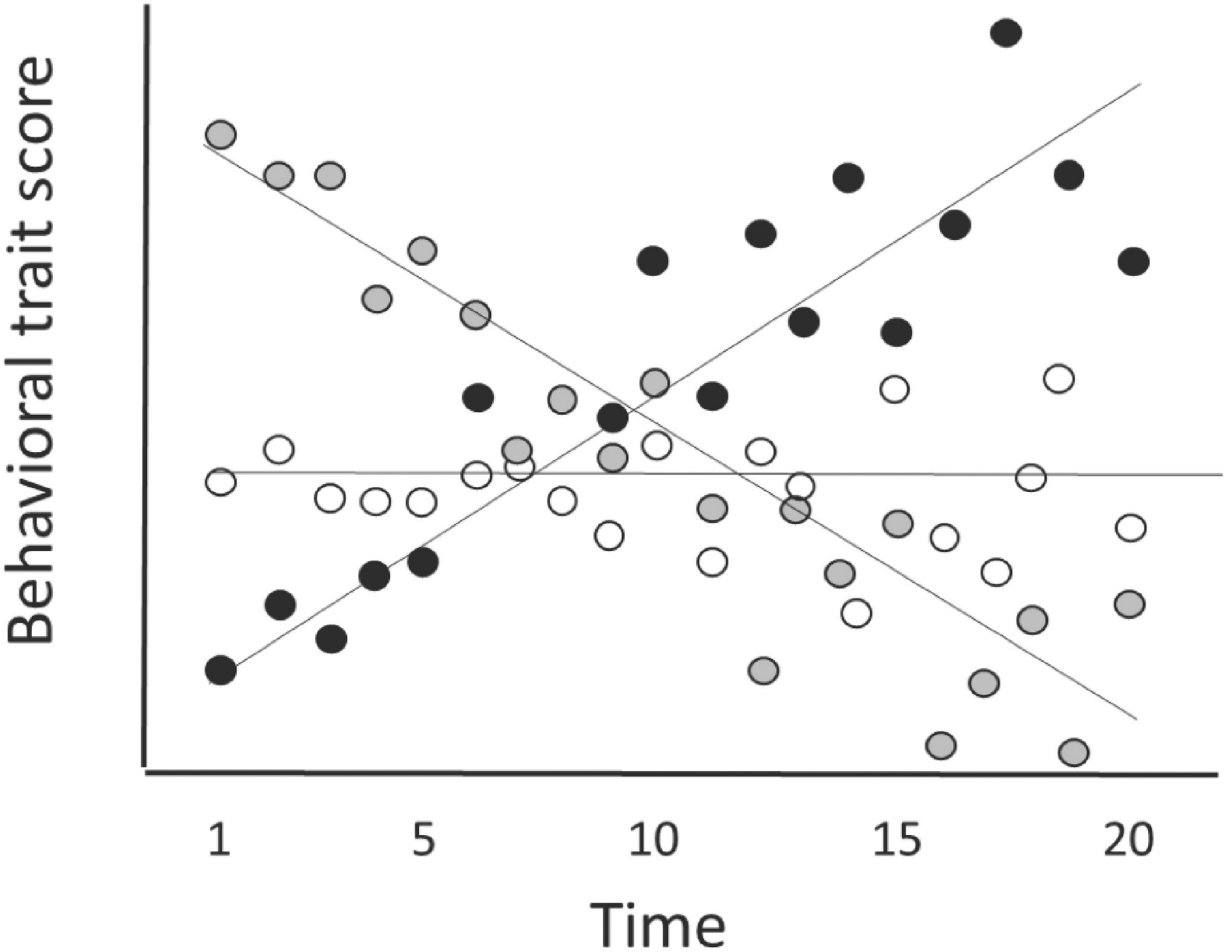
Hypothetical dataset A, collected using a continuous design, showing 20 repeated measures of behavior for each of the three individuals. The scores of each individual at each time point are indicated by dots (black, gray, white), and their expected values at any point in time (i.e., their temporal reaction norms) are indicated by the three lines.

**FIGURE 4:**
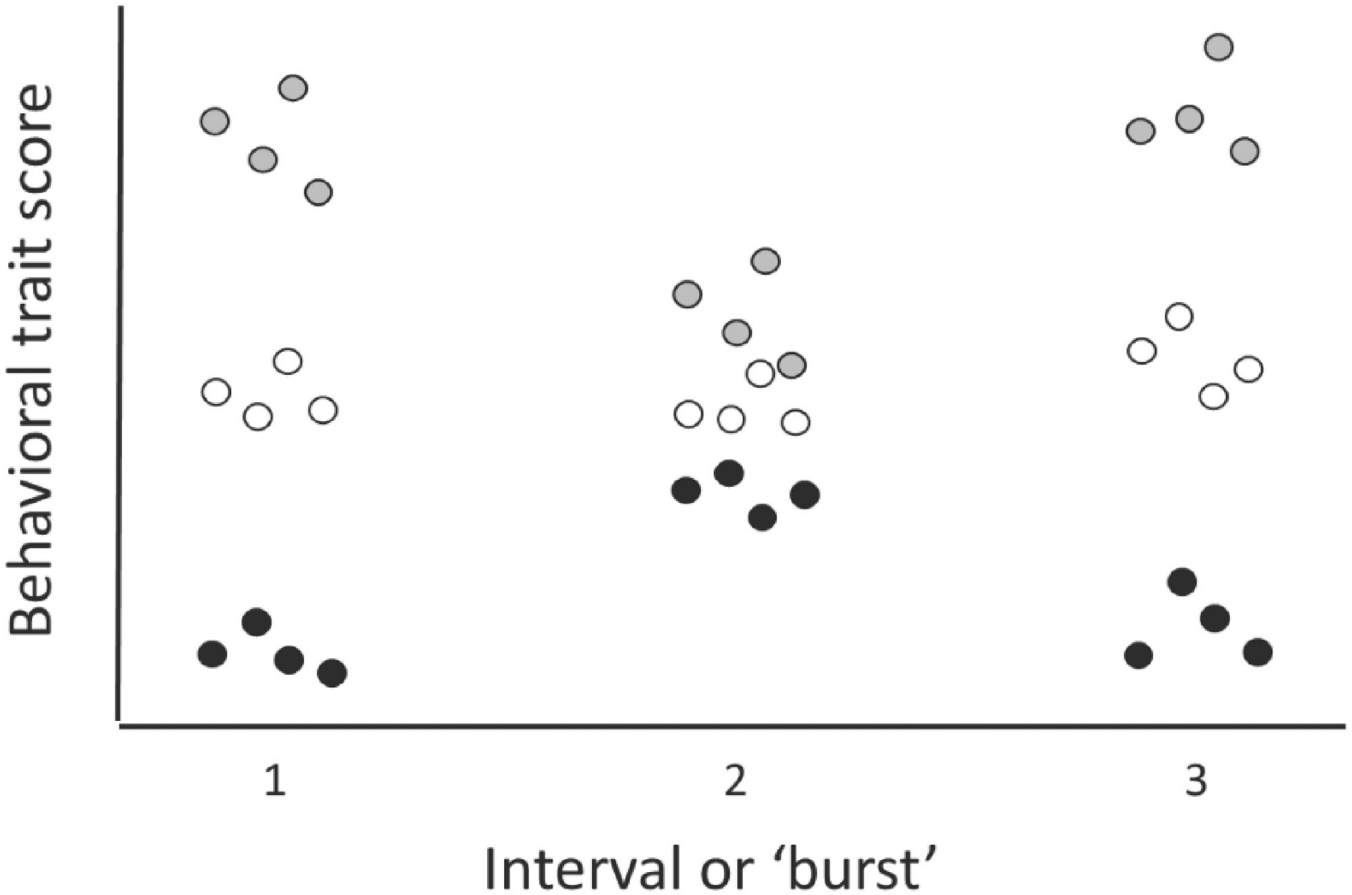
Hypothetical dataset B, collected using a burst design. Each of three individuals in this dataset (indicated by the black, white, and gray dots) was measured four times per burst (e.g., once a day over a 4‐day interval). Each burst was separated by a gap in time from the next one (e.g., data collected at 30‐day intervals).

Because the behavior an individual expresses at a given moment should be viewed as a random sample from an underlying distribution with a mean and variance (Fleeson, 2001; Stamps et al., 2012), it is not advisable to use the first score expressed by an individual to infer its expected value at the beginning of a study. One major problem with this approach is the possibility of “regression to the mean.” That is, if by chance the first datum sampled from an individual's distribution was extremely far from its true mean, we would expect a second datum from that same distribution to be closer to its true mean. These and other statistical issues (e.g., see Beckmann & Biro, 2013) that arise when an individual's first score is used to infer its expected value at the beginning of a study are one reason that the Law of Initial Values, mentioned in the introduction, fell out of favor (Burt & Obradovic, 2013; Rogosa & Willett, 1985). In fact, we should not use each individual's score at any time during a study to estimate its expected behavior at that time, because the residual variation around each individual's expected value is often quite high, as is indicated by the low repeatability of behavioral traits (Beckmann & Biro, 2013; Bell et al., 2009; Wolak et al., 2012). The statistical methods described later in this article avoid the problem of regression to the mean and related issues by estimating each individual's expected values at different points in time based on multiple scores for that individual (e.g., see Figures 3 and 4, below).

Generally speaking, the subjects in empirical studies of convergence or divergence patterns should be of the same age at the onset of the study, since even short‐term temporal changes in behavioral or physiological traits can vary as a function of the age of the subjects. For example, in rats, habituation rates increase over the juvenile to prepubertal period (Leussis & Bolivar, 2006), elevated hormonal levels in response to an acute stressor require twice as long to return to baseline levels in prepubertal individuals as they do for adults (Foilb et al., 2011), and learning rates for a novel spatial learning task decline from middle to old age (D'Hooge & De Deyn, 2001). Moreover, if individuals reach important developmental milestones at different chronological ages, then the subjects should be matched for developmental age, not chronological age. For instance, if conspecifics only begin to direct particular types of aggressive behavior toward focal subjects when the latter begin to approach maturity, and if different individuals in the same species approach maturity at different chronological ages, then any effects of received aggression on the development of the focal subjects' behavior would begin at different chronological ages for the different subjects (Stamps & Luttbeg, 2022). In that case, we would predict that either divergence or convergence in response to those social interactions would begin at a specific life stage (i.e., when each individual approached maturity), as opposed to when they reached a particular chronological age. Finally, if the goal is to study temporal patterns over ontogenetic timescales, the subjects should be as young as is practical at the onset of the study. This is because the theoretical models that predict convergence or divergence patterns over ontogenetic timescales predict that within‐individual changes will be most pronounced when initially naïve subjects are first exposed to salient experiences.

### 3.2 A role for preliminary studies

Designing, conducting, and analyzing experimental studies with the precision required to detect patterns of convergence or divergence is not for the faint of heart, due to the extensive sampling requirements required to obtain robust estimates of the variables of interest (discussed below). Hence, empiricists might first consider some preliminary/pilot analyzes to help them design a given study and indicate whether additional studies of temporal changes in individual differences might be warranted.

Preliminary data can help empiricists determine when to begin and end collecting the data used to test for convergence and divergence patterns, and plan sampling strategies informed by preliminary estimates of among‐ and within‐subjects variation. Answers to these questions will depend on the goal of a given study, and information about the natural history of the study species. For instance, when the goal is to quantify individual differences in habituation or sensitization in response to initially novel stimuli, typically the initial data are collected when the subjects are first exposed to the stimulus (Bell & Peeke, 2012). Similarly, studies of ‘exploratory behavior’ typically begin when subjects are first exposed to a novel object or environment. In contrast, if the goal is to quantify individual differences in activity rates in a familiar environment, then the first data should be taken after all of the subjects had had sufficient time to become familiar with the conditions in their home environment (Biro, 2012).

Information on the biology of the study species is also essential for choosing the appropriate periods over which to measure convergence or divergence patterns. Most of the theoretical models described above assume that each subject is consistently or repeatedly exposed to particular stimuli over the period in which convergence or divergence occur. In nature, however, this assumption might only be valid for particular ages or life stages. For example, Kraft et al. (2018) reported that the tendency of the two male morphs of mosquitofish (*G. holbrooki*) to flee from adult females seemed to gradually converge to a virtually identical score over the adolescent period, but then strongly diverge after the males had reached maturity. These results are consistent with the hypothesis that adult females treated melanic and silver males similarly when they were juveniles, but treated them differently after they reached sexual maturity. Similarly, one would not necessarily expect convergence or divergence patterns to be maintained across other life‐history transitions that resulted in major changes in the physical or social stimuli experienced by a given individual (e.g., metamorphosis, dispersal to new habitat).

Although formal analysis of convergence or divergence requires a longitudinal dataset, preliminary cross‐sectional data may offer some suggestions about the patterns that one might observe in a future study, without requiring a massive commitment of time and resources. For instance, cross‐sectional analyzes of personality traits in humans suggested that time‐specific estimates of among‐individual variance in expected values (VARamg) increase with age (Mottus et al., 2016, 2019), results which the authors interpreted as supporting a divergence pattern. Similar suggestions have been made for animals based on changes in VARamg among samples collected from different life stages or age‐groups (e.g., Petelle et al., 2013; Sakai, 2018). Divergence may also be suspected in experimental studies in which groups of initially similar subjects reared in the presence of different stimuli express different levels of VARamg at the end of this study (e.g., Urszan et al., 2018).

### 3.3 What variables do we need to assess patterns of temporal change?

Our first goal is to verify that significant individual differences in expected values occurred during at least some portion of the study, since otherwise there is no point in asking whether these individual differences changed over time. The typical way to determine whether individual differences occur, or are “repeatable,” is via the statistic *R*. Repeatability (*R*) indicates the proportion of the total variance in scores that is attributable to variance among the subjects in their predicted mean values (VARamg). Of course, many other factors (e.g., time of day, temperature, and reproductive state), can contribute to the total variance in scores in a given dataset, but if the effects of these factors on the scores can be controlled via careful experimental designs and appropriate statistical models, then the total variance in scores will be primarily determined by two variables: VARamg and VARresid, where the latter is the variance that remains after one accounts for variance that can be explained by the other factors. Thus, *R* provides a way to assess the extent to which the variable we are interested in (individual differences in predicted mean values, as is indicated by VARamg) can be detected among the residual noise (VARresid) (Biro & Stamps, 2015). In a carefully controlled study, VARresid can be used to estimate the “predictability” of the subjects, that is, the extent to which their scores varied around their means (Cleasby et al., 2015; Mitchell et al., 2021; Stamps et al., 2012).

There are many reasons why VARresid might vary over time (Biro & Adriaenssens, 2013; Stamps et al., 2012; Westneat et al., 2015), and longitudinal studies have recently confirmed that both VARamg and VARresid can change over time (Biro & Adriaenssens, 2013; Carlson & Tetzlaff, 2020; Class et al., 2019; Cornwell et al., 2023; Kok et al., 2019; Mitchell & Biro, 2017; Polverino et al., 2019; Thys et al., 2021). As a result, temporal changes in either or both of these variables can contribute to changes in *R* over time (reviewed in Dochtermann & Royaute, 2019).

Because we are interested in how individual differences in predicted mean values might change over time, in this article, we seek estimates of time‐specific values of both VARamg and VARresid (VARamg_t_, and VARresid_t_, respectively). Together, these allow us to compute a time‐specific value of *R* (*R*_t_) for each of several different periods within a longitudinal study. The process required to estimate *R*_t_ is slightly more complicated than that required to estimate *R*|time (conditional *R*), a statistic that has often been used to estimate time‐specific *R* (see Appendix S2A). The equations used to compute *R*|time assume that VARamg, but not VARresid, may change over the course of the study (Biro & Stamps, 2015; Nakagawa & Schielzeth, 2010).

By convention, researchers usually assume that consistent individual differences are present if the value of *R* is statistically “significant,” for example, when VARamg (and by extension *R*) is statistically significantly greater than zero, based on a likelihood ratio test when VARamg is evaluated at the intercept (Singer & Willett, 2003), or when the confidence or credible intervals for estimates of *R* are centered away from zero (Biro & Stamps, 2015; Laskowski et al., 2022; Polverino et al., 2016). Hence, the first criterion for any study of temporal changes in individual differences is that the value of *R*_t_ must be significant for at least one of the periods over the course of a longer study. Of course, given a sufficiently powerful experiment, even very low values of *R* may be significant. Thus, some researchers might prefer to set the bar a bit higher, and require that the value of *R*_t_ should reach some threshold value (e.g., a ‘moderate’ effect size of 0.3, see Cohen, 1988) at some point during a longer study to justify taking a closer look at temporal changes in individual differences over the course of that study.

Assuming that individual differences were observed at some point in the study, our next question is whether, and if so when, the expected values of the subjects became more similar to one another (convergence), more different from one another (divergence), or were maintained at the same level over time (maintenance), during a specific period of time during the course of a longer study. In order to address this question, we need to graph the raw data, ensure that our statistical model captures the patterns in that data, and then use that model to estimate the value of several time‐specific variables: VARamg_t_, CORR_t1,t2_, and CORRe_t_,s. The equations used to compute these variables are standard variance partitioning exercises developed in the quantitative genetics literature, but they are unfamiliar to many behavioral biologists, and they are currently scattered among a number of publications (e.g., Brommer, 2013; Falconer, 1981; Mitchell & Houslay, 2021). Here, we bring these formulae together to show how they can be used to determine whether and when individual differences change over time. To this end, we provide simple explanations of two types of statistical models that can be used to estimate these variables, detailed step‐by‐step worked examples based on published datasets, and annotated code which empiricists can use to analyze their own data (see below and Appendices S1–S4). Our goal is to introduce readers to two classes of statistical models that can be used to describe changes in individual differences over time, equations that can be used to estimate time‐specific variances and covariances, and ways that different models with different assumptions can be used to analyze data on temporal changes in individual differences.

1. VARamg_t_ This is the variance among the subjects in their expected (predicted mean) values at a given time, *t*. A decline in VARamg_t_ during a given period suggests that convergence occurred during that period. Conversely, an increase in VARamg_t_ during a given period suggests that divergence occurred during that period. Finally, maintenance of VARamg_t_ over a given period suggests that differences among the subjects in their expected values were maintained over that period.
2. CORR_t1,t2_. This is the correlation, across subjects, between the estimates of their expected values at times t1 and t2. This correlation allows us to determine whether rank‐order consistency was maintained t1 and t2. Rank‐order consistency is important because it indicates the extent to which individual differences were maintained over time on an ordinal scale, without regard to the extent to which the predicted scores of the subjects differed from one another (see Roberts & DelVecchio, 2000; Stamps & Groothuis, 2010). CORR_t1,t2_ will be positive if consistency is maintained over the period between time t1 and t2, negative if the order of the subjects' scores reversed between time t1 and t2, and near zero if consistency was not maintained over the interval t1 to t2. We suggest that positive or negative values of CORR_t1,t2_ approach “moderate” effect sizes (e.g., *r* ≥ 0.3 or *r* ≤ −0.3, Cohen, 1988), to increase the chances that rank‐order consistency is biologically, as well as statistically, significant. On a graph showing the subjects' reaction norms, CORR_t1,t2_ is indicated by the extent of crossing‐over that occurred during the period between t1 and t2, such that higher levels of crossing‐over yield lower values of CORR_t1,t2_. This correlation is similar to an intra‐class correlation or repeatability estimate, but it is based on the subjects' expected scores, rather than on their raw scores, as is the case for the latter statistics.
3. CORRe_t_,s. This is the covariance across subjects, between their ‘elevation’ (i.e., the estimate of their expected value at a given time, *t*), and their “slope,” that is, the rate of change in their expected values after time *t*, expressed as a correlation. If our time variable is left centered (see below), then the covariance between intercepts and slopes, expressed as a correlation, is indicated by CORRe_0_,s. The CORRe_t_,s will be negative if the mean values of the subjects converged after time *t*, positive if their expected values diverged after time *t*, and near zero if differences in their expected values were maintained after time *t*. Here too, we suggest using “moderate” effect sizes described above as support for substantive and biologically relevant correlations.

Crucially, none of these variables on its own may be sufficient to tell us whether individual differences converged, diverged, or were maintained during a particular period of time. For instance, although a positive value of CORRe_t_,s indicates divergence, divergence could also occur if CORRe_t_,s was near zero. The latter situation would be expected if all of the subjects started out with similar expected values at t1, but diverged to very different expected values by t2. In this case, the low variance in expected values at t1 would lead to low values not only of CORRe_1_,s, but also of CORR_t1,t2_. However, the divergence would still be apparent, based on a substantial increase in VARamg_t_ from t1 to t2, and a “fanning out” pattern in a graph that illustrated the subjects' temporal reaction norms during this period.

Along the same lines, although similar values of VARamg_t_ at t1 and t2 might suggest maintenance, this could also occur if substantial crossing‐over of the subject's temporal reaction norms occurred between t1 and t2. At the extreme, the trait values of the different subjects might even reverse, such that individuals with high expected values at t1 had low expected values at t2, and vice versa (e.g., of “reversal patterns,” see Figure 3, and figure 2d in Brommer & Class, 2015). However, in the latter situation, CORRe_1,s_ and CORR_t1,t2_ would both be negative and the crossing‐over would be obvious in a graph illustrating the reaction norms of the subjects.

As we demonstrate below, by graphing the subjects' data and computing the values of all of the time‐specific variables described above, empiricists can determine whether individual differences increased, decreased, or were maintained during each of several periods during a longer study.

### 3.4 Experimental designs

Temporal changes in individual differences can be analyzed using different types of longitudinal experimental designs. These days, empiricists typically use one of two longitudinal designs to describe temporal trends in individual differences: (1) continuous designs, or (2) burst designs. In a continuous design, the observations for each of the subjects are relatively evenly spaced apart in time over the course of the study period. In contrast, in a burst design, a series of observations are closely spaced in time, with gaps between each “burst” of data collection (Nesselroade, 1991; Salthouse & Nesselroade, 2010).

One advantage of the continuous design is that investigators do not need to decide, a priori, when they should focus on data collection. In contrast, the burst design is useful when investigators begin the study with an idea of the periods for which they require robust estimates of individual differences (e.g., morning vs. evening, juvenile vs. adult life stages, behavior expressed at the onset of each breeding period). In addition, as we describe below, data collected using a burst design can be analyzed using a statistical model (the discrete time model) which relies on fewer assumptions than does another model (random regression) which is often used to analyze convergence or divergence patterns. Below, we illustrate time‐specific convergence and divergence using hypothetical datasets collected using the continuous design (Figure 3) and the burst design (Figure 4).

Visual inspection of the hypothetical continuous dataset presented in Figure 3, suggests that it illustrates a “reversal pattern,” in which the rank‐order of the subjects switched over the course of the study period. For instance, one subject (black dots) appeared to have a relatively low expected value at the onset of the study, but ended up with a relatively high one, while the reverse was true for another subject (gray dots). In this situation, we would expect CORR_t1,t2_ to change over the course of the study period, with positive values for CORR_1,5_ and for CORR_15,20_, but negative values for CORR_1,20_. In addition, we would expect VARamg_t_ to decline early in the study, and then increase later in the study, and we would expect CORRe_t_,s to be negative early in the study, approach zero around day 9, and then become increasingly positive with time. This figure also suggests that residual variance (VARresid_t_) increased over time, as is indicated by the deviations of the subjects' scores from their temporal reaction norms; these deviations appear to be larger later than earlier in the study.

Visual inspection of the hypothetical burst data in Figure 4 not only suggests that the individuals had different expected values within each burst, but also that the rank‐order consistency in their expected values was maintained across the study period. In that situation, we would expect both CORR_1,2_ and CORR_1,3_ to be positive. This figure also suggests that the variance among the subjects in their expected values (VARamg_t_) decreased from burst 1 to burst 2, but then increased again from burst 2 to burst 3. In addition, the figure suggests that convergence occurred between burst 1 and 2, but that divergence occurred between burst 2 and 3. In that case, we would expect CORRe_t_,s to be negative over the period from t1 to t2, but positive over the period from t2 to t3.

Close visual inspection of the raw data of the subjects of a given study is not only required to appropriately fit statistical models, but also to interpret their results. While this might be obvious to many, empiricists studying individual differences often seem to proceed directly to analysis without plotting data, and many of them fail to provide the plots of a model's predictions against the raw data that would allow readers to evaluate the authors' conclusions for themselves. To this end, in Appendices S1–S4, we provide the code and analyzes required to determine whether a given statistical model captures trends evident in graphs of the raw data of the individuals in the study, and whether the data satisfies the basic assumptions of that statistical model.

## 4 STATISTICAL MODELS FOR ANALYZING TIME‐SPECIFIC CONVERGENCE OR DIVERGENCE

### 4.1 Continuous time analysis using random regression

#### 4.1.1 General approach

Researchers studying temporal trends in longitudinal studies in psychology (Singer & Willett, 2003) and behavioral ecology (Dingemanse et al., 2010) often employ the familiar random regression (RR) model. In its simplest form, this model assumes that one can characterize the temporal trendlines of all of the subjects using straight lines about a linear mean level trend, for example, that if convergence or divergence do occur, these patterns are maintained over the entire course of a study (see Figure 1a,b). In more complex forms, the RR may take on nonlinear patterns at the mean level trend by treating time as a factor (or using polynomial terms), but the individual trendlines are still assumed to be linear deviations about the mean (more details below). As such, the simple RR model provides a useful starting point for modeling change over time, because it can capture some of the possible patterns of temporal change (see Figure 1). In addition, it provides a null model that can be rejected for more complex patterns (see Figure 2), given a sufficiently powerful dataset and a statistical approach that is capable of detecting changes in temporal trends over time.

We suggest that the RR model provides a useful starting point for analyses of temporal changes in individual differences in predicted mean values, because as a practical matter, empiricists often do not know at the onset of a study if or when the trendlines of their subjects might change over time. For instance, in a typically noisy empirical dataset, it might not be obvious a priori whether the data best conformed to the pattern illustrated in Figure 1a, or to the pattern illustrated in Figure 2a. Here, we show how time‐specific variables generated by an RR analysis can be used to suggest whether or not convergence or divergence patterns might have occurred during particular periods over the course of a larger study. In such cases, we suggest how empiricists might collect additional data and use more‐complex statistical models to pin down the periods when convergence or divergence, if present, occurred (see below, and Section 5).

In brief, a random regression model does two things. First, it describes the mean trendline for the subjects (i.e., the trendline for the population) much as the familiar linear regression model does. Second, it characterizes how the trendline of each subject differs from this population mean trendline. The intercept and slope parameters for the population are fixed effects, while the predicted intercepts for each subject and the predicted slopes for each subject are characterized by random effects, each of which is expressed as a deviation from the population‐level intercept and slope. For an introduction to this model, we recommend reading from textbooks on the subject (Singer & Willett, 2003; Zuur et al., 2009), but we also provide a brief review of relevant models and code here, to help readers understand and implement them.

For studies of temporal changes in individual differences, the intercept should be defined at the point in time when the first data were collected (see Singer & Willett, 2003 for discussion of data centering in longitudinal models). That is, where time is indicated by *t*, the time when the first data were collected is set as *t* = 0, by subtracting the minimum time value from all time values. This is referred to as “left centered” data. This practice differs from other situations in which temporal change in trait values is not the focus, and the intercept is set at the temporal midpoint of the study (i.e., “mean centered,” e.g., as in Dingemanse et al., 2010).

Together, the predicted intercepts and slopes for each subject from the random regression define the initial expected value and how each subject's expected values changed over time, respectively, thus providing an estimate of each subject's temporal reaction norm. Using the equations described below, and model estimates of the among subjects' variance in intercepts and slopes, and their correlation, allow us to estimate each subject's expected value (its “elevation”) at any time, *t*, during the study. This permits us to estimate VARamg_t_ at any point in time. Similarly, we can compute the correlation, across the subjects, between their expected values at any two points of time during the study (CORR_t1,t2_), in order to determine the extent to which rank‐order differences in trait values were maintained over specific intervals over the course of the study. Finally, the estimates of the subjects' elevations at specific times, combined with the estimates of their slopes, allows us to estimate CORRe_t_,s for any time during the study. Since the intercept is left centered at the onset of the study, CORRe_0_s = CORR_i,s_ where CORR_i,s_ is the correlation, among the subjects, between their intercepts and slopes. Together, estimates of CORR_t1,t2_, VARamg_t_, and CORRe_t_,s, at different points over the course of the study can indicate whether the rank‐order consistency of the different subjects was maintained (and if so, when during the study it was maintained), and whether convergence or divergence occurred (and if so, when during the study it occurred). Finally, time‐specific estimates of repeatability (*R*_t_), based on estimates of VARamg_t_ and VARresid_t_, can be used to estimate the extent to which subjects differed from one another at different times over the course of the study.

#### 4.1.2 A worked example

We used data from Jolles et al. (2019) to demonstrate how a random regression model can be used to analyze an existing dataset produced using a continuous time experimental design. Jolles et al. (2019) investigated temporal changes in “boldness” (based on an assay of the proportion of time spent out of shelter in an initially novel test tank) of first‐year, three‐spined sticklebacks, *Gasterosteus aculeatus*. Each of the subjects was tested once a week, for 6 consecutive weeks. The resulting dataset was suitable for analysis using the random regression model, as it had a sample size (80 subjects, six repeats per subject) sufficient to estimate model parameters with reasonable precision (see van de Pol, 2012 and Martin et al., 2011 for discussion of the sample sizes required for this sort of analysis). A step‐by‐step description of our analysis of these data is presented in Appendix S2A, using code provided in Appendix S1.

**FIGURE 5:**
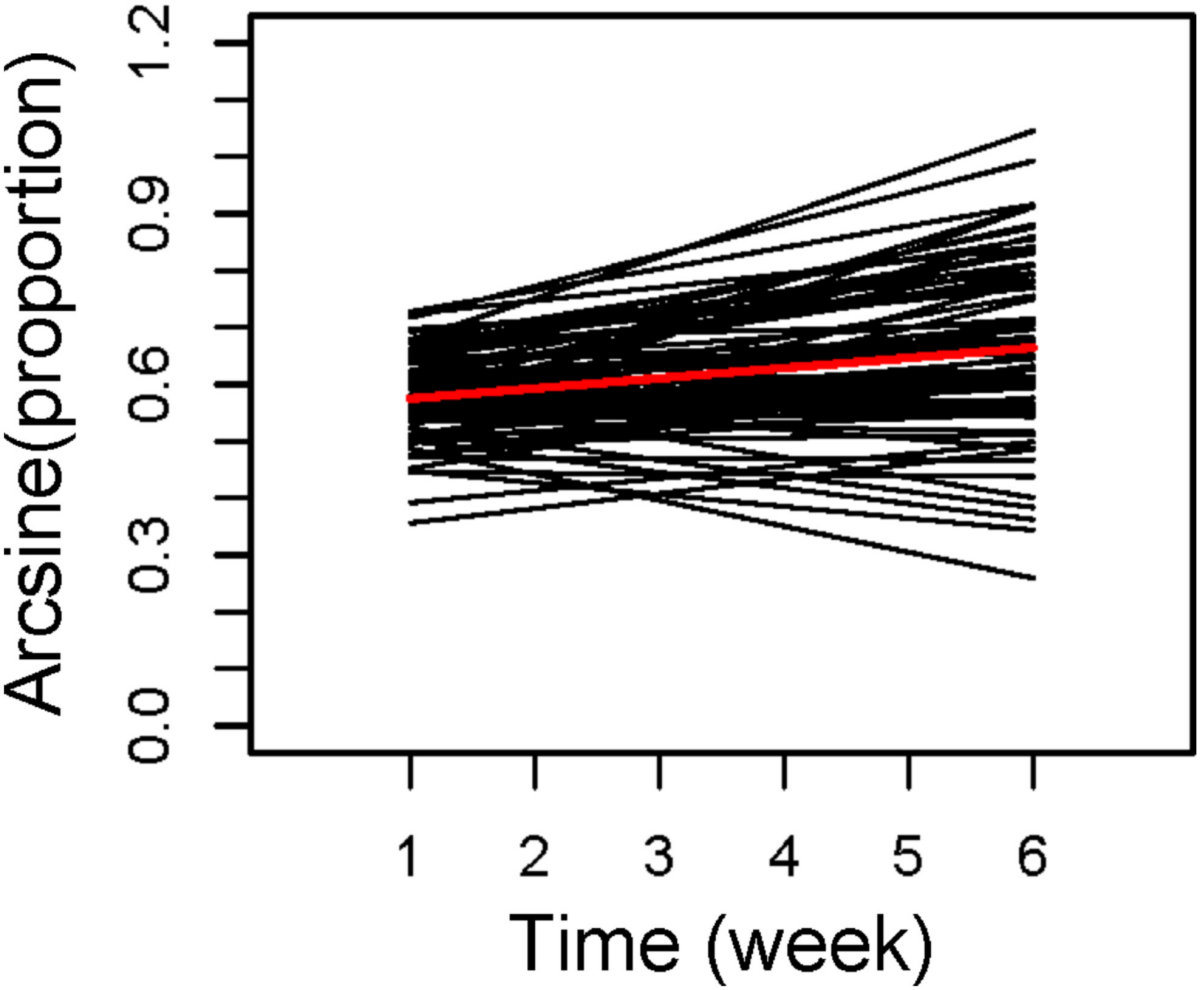
Predicted temporal reaction norms for the subjects in Jolles et al. (2019), generated by a random regression model that permits residual variance to vary over time. Black lines indicate the predicted reaction norms for each subject, and the red line indicates the mean level trend. Results based on code provided in Appendix S1 and analyzes described in Appendix S2.

The results of this analysis suggested that individual differences in boldness in the stickleback were rather similar and largely maintained for the first week or two, and were then followed by divergence that began in week 3 and continued to week 6 (see Table 1, Figure 5, and Appendix S2A). The suggestion of maintenance in weeks 1 and 2 is supported by the very similar values (and 95%CI's) of VARamg_t_ in weeks 1 and 2, and estimates of CORRe_t_,s that broadly overlapped zero in both periods. Divergence later in the study was indicated by increases over time in the value of VARamg_t_, and significantly positive values of CORRe_t_,s from week 3 onward. Rank‐order consistency was somewhat maintained from weeks 1 through 6, but was lower earlier (weeks 1–3) than later (weeks 4–6) in the study (based on analyzes of CORR_t1,t2_). Individual differences in mean values were also evident throughout the study, but were much less pronounced earlier than later (results from the *R*_t_ analysis). The uncertainty in the estimates of the value of CORRe_t_,s and the low values of *R*_t_ in weeks 1 and 2 were at least partly attributable to the fact that VARresid_t_ was significantly and substantially higher during weeks 1 and 2 than it was later in the study. This trend was apparent in the raw data (see Appendix S2: Figure A2.2) and it was supported by analyzes which showed that adding time‐specific residual variance to a model substantially improved the fit of the model to the data (see Appendix S2B).

**TABLE 1:** Estimates for VARamg_t_, VARresid_t_, CORR_t1,t2_, CORRe_t_,s, and *R*_t_ presented for each time point derived from random regression analysis of the behavioral data from Jolles et al. (2019). Mean and CIs are indicated for each variable. Results from code provided in Appendix S1, and from analyzes described in Appendix S2A.

Week	A	B	C	D	E
	VARamg_t_	VARresid_t_	CORR_1,X_	CORRe_t_,s	*R*_t_
1	0.0109 (0.005 to 0.019)	0.028 (0.018 to 0.041)	NA	−0.18 (−0.52 to 0.38)	0.28 (0.14 to 0.44)
2	0.0105 (0.006 to 0.016)	0.019 (0.013 to 0.027)	0.95 (0.90 to 0.98)	0.12 (−0.27 to 0.58)	0.36 (0.22 to 0.51)
3	0.0121 (0.008 to 0.018)	0.009 (0.006 to 0.014)	0.82 (0.67 to 0.93)	0.39 (0.04 to 0.73)	0.56 (0.42 to 0.70)
4	0.0158 (0.01 to 0.023)	0.009 (0.007 to 0.014)	0.66 (0.42 to 0.87)	0.59 (0.31 to 0.83)	0.61 (0.48 to 0.73)
5	0.0220 (0.015 to 0.031)	0.008 (0.005 to 0.013)	0.52 (0.21 to 0.81)	0.72 (0.51 to 0.88)	0.72 (0.59 to 0.84)
6	0.0290 (0.020 to 0.043)	0.011 (0.006 to 0.018)	0.41 (0.07 to 0.76)	0.81 (0.64 to 0.92)	0.72 (0.58 to 0.85)

The results of this model, which computed estimates of VARresid_t_ for each week, were substantially different (especially for the first 2 weeks) from those of an otherwise equivalent random regression model which assumed that residual variation did not change over time (see Appendix S2B). Moreover, the patterns indicated by the current model were also substantially different from those reported by Jolles et al. in their analyzes of their data. Jolles et al. (2019) relied on a random regression model that assumed that residual variance did not change across the study period, and that estimated their subjects' intercepts at week 0. They reported a highly significant negative correlation across the subjects between their intercepts and their slopes (CORR_i,s_ = −0.56). Taken on its own, this result would either be interpreted as evidence of convergence throughout the study period, or of a reversal pattern, in which an initial period of convergence was followed by a later period of divergence. In contrast, our model provided no evidence of convergence at any time, and instead suggested that an initial period of maintenance was followed by a period of divergence. Confirming these results would require additional study and other statistical models, some of which are described below and in Section 5.

### 4.2 Burst sampling designs analyzed using discrete time models

#### 4.2.1 General approach

Data collected using a burst design can be analyzed several different ways. The first is to use a random regression model in which time is treated as a categorical, rather than a continuous variable (a “categorical time model,” e.g., Class et al., 2019; Dingemanse, Barber, et al., 2012). This type of model allows for nonlinear trendlines at the population level, and by extension at the individual level. However, this model still assumes that each individual's deviation from the population‐level mean at each burst is linear as a function of time, meaning that every individual's predicted trendline has a similar shape (see Appendix S4B). Another option for burst data is to use a “discrete time model,” sometimes referred to as a “character state” model. An advantage of this model is that it makes no assumptions about deviations of the individuals' reaction norms from the reaction norm at the population level, and thus the shapes of the temporal reaction norms are allowed to vary among individuals (as in Figure 2). The discrete time model requires more parameters than does the categorical time model. However, given adequate data, the discrete time model may be preferable for analyzing burst data, because it allows for the possibility of complex patterns of temporal changes in variances that theory suggests may occur over extended periods of time (see Section 1). Hence, in this section, we highlight the discrete time model. Furthermore, when we analyzed our worked example dataset (Mitchell et al., 2016, see below), using both types of models, we found that the discrete time model provided a better fit to these data than did the categorical time random regression model (see Appendix S3 for code, and Appendix S4B for a comparison of results from the two models).

#### 4.2.2 A worked example

We used data from Mitchell et al. (2016) for a worked example to demonstrate how an existing dataset that was collected using a burst design could be analyzed using a discrete time model. In this study, adult male guppies were placed in individual home tanks, and their activity in those tanks was measured over a 3‐week period. This dataset was selected for analysis because the data were collected using a burst design (each subject was sampled 4–6 times per burst, over 2–3 days, in three bursts conducted at weekly intervals), and because the sample size (104 individuals, total *N* = 1477) was adequate for this type of analysis.

**FIGURE 6:**
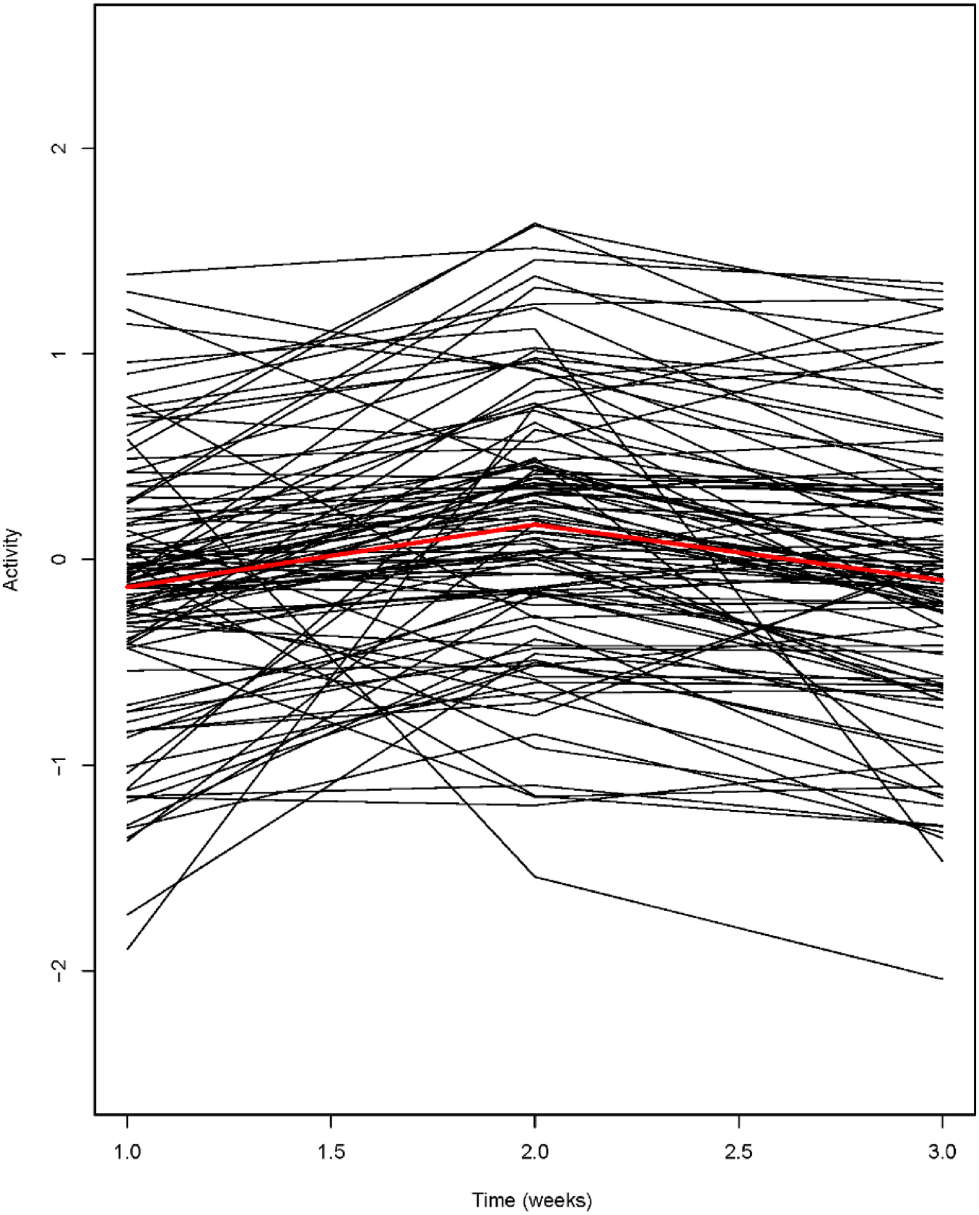
Fitted temporal trendlines of guppy activity rates from Mitchell et al. (2016), based on three bursts of data, analyzed using a discrete time (“character state”) type model which permits among‐subjects variance and residual variation to vary over time. Shown are 104 individuals, with the mean level trend shown in red. Activity is expressed in units of SD following transformation. Results based on code provided in Appendix S3, and analyzes described in Appendix S4A.

The results of our analysis of the data in Mitchell et al. (2016) indicated that moderate convergence in activity occurred between week 1 and week 2, followed by weak, if any, convergence between week 2 and 3 (Table 2, Figure 6, Appendix S4A). These results were based on weak CORR_1,2_ and a significantly negative value of CORRe_t_,s from week 1 to 2, compared to strong CORR_2,3_ and a weakly negative, close to nonsignificant, value of CORRe_t_,s from week 2 to 3 (Table 2). Note, however, that in this dataset, convergence was not accompanied by substantial changes in VARamg_t_ across the three bursts. CORR_t1t2_ values were significantly higher than zero across the study, indicating maintenance of rank‐order consistency throughout. However, CORR_1,2_ was somewhat lower than CORR_2,3_, reflecting the higher levels of crossing‐over of the subjects' reaction norms that occurred earlier than later in the study (Figure 6).

**TABLE 2:** Estimates for VARamg_t_, VARresid_t_, CORR_t1,t2_, and *R*_t_ presented for different time points, based on discrete time and random regression analyses of data from Mitchell et al. (2016). Mean and CIs are indicated for each variable. Results based on code provided in Appendix S3, and analyzes described in Appendix S4A.

Week (X)	A	B	C	D	E
	VARamg_t_	VARresid_t_	CORR_1,X_	CORR_2,3_	*R*_t_
1	0.56 (0.39 to 0.80)	0.55 (0.46 to 0.64)	NA		0.50 (0.40 to 0.61)
2	0.50 (0.37 to 0.67)	0.36 (0.31 to 0.41)	0.42 (0.22 to 0.60)		0.58 (0.50 to 0.66)
3	0.56 (0.39 to 0.76)	0.53 (0.47 to 0.61)	0.26 (0.04 to 0.47)	0.69 (0.53 to 0.81)	0.51 (0.41 to 0.60)

## 5 DISCUSSION

Over the years, investigators working in a wide range of disciplines, including physiology, ethology, psychology, and behavioral ecology, have described situations in which individual differences in behavioral or physiological traits seem to increase (diverge) or decrease (converge) during particular periods over the course of longer study. These observations have encouraged theoreticians to advance an impressive range of hypotheses that predict that individual differences will either diverge or converge during specific periods of time. The ability to identify when convergence or divergence patterns occur, begin, or cease is a crucial first step for evaluating the proximal or the ultimate factors that might be responsible for generating these patterns. However, to date, empiricists have rarely quantified time‐specific convergence or divergence patterns, in part because of a lack of appreciation of experimental designs and statistical methods which would allow them to do so.

Here, we show how estimates of several time‐specific statistics allow investigators to quantify changes in individual differences over time. These include VARamg_t_, the variance among individuals in their expected values at a given time, CORRe_t_,s, the correlation, across individuals, between their expected values (i.e., their “elevation”) at a given time and the rate of change in their expected values after that point in time (i.e., their “slope”), and CORR_t1,t2_, the correlation, across individuals, between their expected values at t1 and their expected values at a later time, t2. We describe how estimates of these three variables, together with graphs illustrating the temporal reaction norms of the subjects, can suggest if, and when, individual differences in expected (predicted mean) values converged, diverged, or were maintained in each of a series of periods during a longer study. Estimates of the time‐specific residual (unexplained) variance at each time period, VARresid_t_, are also required, because if the residual variance changes over time, failing to account for those changes can bias estimates of the other variables listed above (Ramakers et al., 2020). Finally, investigators can use the time‐specific values of VARamg_t_ and VARresid_t_ to estimate *R*_t_ (time‐specific values of repeatability), in order to estimate the extent to which individual differences changed over time.

Because at present the statistical methods suitable for analyzing time‐specific changes in individual differences are unfamiliar to many of the empiricists who might want to use them, in this article, we provide two worked examples of these methods, both of which are based on published data from laboratory studies of fish behavior. In the first example, Jolles et al. (2019) assessed “boldness” in three‐spined stickleback (*G. aculeatus*) once a week for a total of 6 weeks. In this case, a random regression model suggested that individual differences in boldness were largely maintained during the first 2 weeks of the study, and that divergence began at week 3 and continued through the end of the study. In the second example, Mitchell et al. (2016) recorded the activity of male guppies (*P. reticulata*) in three bursts, 1‐week apart. In this case, a discrete‐time model indicated that the mean activity rates of the subjects strongly converged from week 1 to week 2, then weakly converged from week 2 to week 3. If nothing else, these examples show that different patterns of time‐specific change in behavior can occur even in empirical studies which are superficially similar (temporal changes in “personality” traits of fish measured over several weeks in an initially novel environment), for reasons which are currently unclear.

More generally, these worked examples show why estimates of all of the time‐specific statistics described in this article can be important for detecting and describing temporal changes in individual differences. For instance, many investigators have described convergence or divergence patterns using statistical models which assume that VARresid does not change over time (e.g., Bell & Peeke, 2012; Biro et al., 2014; Jolles et al., 2019; Martin & Reale, 2008; Mathot et al., 2012). However, theoreticians have shown that statistical models which either do or do not allow VARresid to vary over time can produce different results (e.g., Ramakers et al., 2020). Hence, we analyzed Jolles' stickleback data both ways (see Appendix S2B). For that dataset, random regression models which assumed that VARresid did not change over time (including the model Jolles used to analyze their data) reported strong negative correlations between the subjects' estimated values at the onset of the study and their slopes. Typically, this result would be construed as evidence for either convergence, or reversal (i.e., in which a period of convergence was followed by a period of divergence). In contrast, our model, which included time‐specific estimates of VARresid, instead suggested that an initial period of maintenance was followed by a period of divergence, results which were supported by visual inspection of the temporal reaction norms of the experimental subjects.

Similarly, investigators who have tested for temporal changes in individual differences using random regression models typically estimate the correlation between elevation and slope (CORRe_t_s) at just one point in time (e.g., Beveridge et al., 2022; Biro et al., 2014; Class & Brommer, 2016; Dingemanse, Bouwman, et al., 2012; Martin & Reale, 2008; Mathot et al., 2012; Thys et al., 2021). This practice might be adequate for detecting convergence or divergence if either of those patterns was sustained from the beginning to the end of the study (e.g., patterns indicated in Figure 1). However, as we have shown here for the stickleback analysis, if we had just relied on a single estimate of CORRe_t_s, our conclusions about temporal changes in individual differences would have varied, depending on when we estimated the relationship between the elevation and the slope. For instance, if we had estimated this correlation using estimates of the subjects' expected values at the onset of the study, we would have concluded that there was no evidence for either convergence or divergence, whereas if we had estimated the correlation based on estimates of the subject's expected values at the midpoint of the study, we would have concluded that the individual trendlines diverged throughout the study.

Other investigators have often relied on just one of the time‐specific statistics mentioned above to determine whether convergence or divergence occurred in their study. For example, several authors have used changes in VARamg with age as evidence of temporal changes in individual differences (Mottus et al., 2017; Petelle et al., 2013; Sakai, 2018). But as we demonstrate here with the analyzes of the guppy data from Mitchell et al. (2016), convergence (indicated by negative values of CORRe_t_s) can occur even in the absence of major temporal changes in VARamg when there is substantial crossing of the subjects' temporal reaction norms.

Of course, both the worked examples in this article were meant to be illustrative, showing what different models can and cannot do when visual inspection of a dataset indicates some obvious trends that guide analyzes. In the future, research in which simulations were used to create complex datasets with known underlying structures would be valuable for evaluating and comparing the performance of different models in capturing temporal trends over a wide range of conditions, including variation in the heterogeneity of residuals (see also below).

One unexpected insight to emerge from our review was that to date, researchers have relied on different criteria to determine when individual differences (or “personality”) emerge over the course of development. The theoretical models described in the Introduction predict temporal changes in the true (as opposed to the predicted) means of the subjects. In these models, the emergence of individual differences is assumed to be a product of divergence, whereby individuals who had very similar mean values at one point in time gradually diverge until their mean values are quite different from one another (e.g., see Bergmuller & Taborsky, 2010; Fisher et al., 2018; Sih et al., 2015). However, empiricists usually describe the emergence of individual differences in practical terms, based on the time or age at which they are first able to detect individual differences in trait values, using repeatability, *R* (e.g., Brust et al., 2015; Laskowski et al., 2022; Polverino et al., 2016). Because empiricists rely on statistically significant values of *R* to detect individual differences in predicted mean values, and because the ability to detect statistically significant time‐specific values of *R* (*R*_t_) depends upon the sample size and the values of both VARamg_t_ and VARresid_t_, the age or time when individual differences “emerge” based on this second criterion will depend on how both VARamg_t_ and VARresid_t_ change over time. As a result, individual differences could “emerge” at a given age or time in an empirical study even in the complete absence of any divergence in predicted mean values. For instance, Polverino et al. (2016) found that the *R* values for several personality traits in mosquitofish (*G. holbrooki*), were significant for adults but not for juveniles, not because VARamg_t_ changed over ontogeny, but because VARresid_t_ declined with age. Thus, the inability to detect individual differences in predicted mean values prior to a given point in time during a longer study could occur because VARamg_t_ was very low before that point, because VARresid_t_ was very high until that point, or some combination of these. The best way to discriminate among these alternatives would be to increase sampling efforts to estimate parameters with greater precision (see simulations of data requirements in Martin et al., 2011; van de Pol, 2012; Wolak et al., 2012). For example, by sampling each subject's behavior at 3 s intervals for 11 h per day, Laskowski et al. (2022) were able to demonstrate individual differences in the mean swimming speed of individual fish, *Poecilia formosa*, within the first day post‐hatch (*R*_t_ = 0.65). Note, however, that such frequent sampling might produce inflated estimates of VARamg_t_ (and, by extension, *R*_t_) if individual scores were strongly autocorrelated over short periods of time. Although methods currently exist to estimate temporal autocorrelation, they do not take into account the possibility that temporal autocorrelation might differ among individuals.

Our initial reason for estimating time‐specific values of VARresid was to obtain more reliable estimates of other variables required to test for temporal changes in individual differences. However, this procedure indicated that VARresid_t_ for boldness significantly declined over time in the stickleback (see Table 1), but that VARresid_t_ for activity rates did not change over time in the guppies (see Table 2). In both studies, the investigators controlled experimentally and statistically for other factors that might have contributed to variation in the subject's behavior; hence, we assume that much of the residual variance observed in each study was due to rIIV (residual intraindividual variability, or its inverse, “predictability”). Further, in both of these studies, the fish were repeatedly tested in the same environment, under the same set of conditions. In this situation, Bayesian models of development predict that rIIV will decline as a function of time (e.g., Stamps & Krishnan, 2014a). This is because as the subjects become increasingly certain over time about the true current value of the state of the environment, they become more certain about the trait values that would be appropriate for that environment. And to date, empiricists who have studied temporal changes in predictability in this situation often find that rIIV declines (or equivalently, that predictability increases) as a function of time, age, or the number of experiences. For instance, declines in trial‐to‐trial variability as a function of time or practice sessions are frequently reported in studies of motor learning (Beerse et al., 2020; Krakauer et al., 2019; Shmuelof et al., 2012, reviewed in Sternad, 2018). Declines in rIIV over time have also been reported for other behaviors when the subjects are repeatedly tested under the same conditions (Biro & Adriaenssens, 2013; Goold & Newberry, 2017; Mitchell & Biro, 2017; Polverino et al., 2019; Thys et al., 2021). Hence, the decline in VARresid_t_ over time in boldness we detected in the stickleback is in line with both theoretical predictions and previous empirical studies of temporal changes in this variable when animals are repeatedly or continuously exposed to the same stimuli or experiences.

Although we illustrated the methods used in this article using two datasets which focused on a similar situation (changes in individual differences in fish behavior over several weeks for subjects tested in an initially novel environment), the methods we describe here should be useful for analyzing many other situations in which convergence or divergence might be restricted to particular periods of time. For example, discrete time models might be used to analyze situations in which preliminary results suggest that convergence and divergence patterns might regularly alternate over time. One possible example is described by Kluen and Brommer (2013), which measured “neophobia‐related behavior” in blue tits (*Cyanistes caeruleus*) in two seasons: winter and breeding season. These authors found that the individuals had significantly different scores in winter, but that their scores converged to very similar values during the breeding season. The low variance among the subjects in expected mean values during the breeding season likely contributed to results indicating low rank‐order consistency across the seasons (e.g., values of CORR_t1,t2_ near zero). These results suggest that in blue tits, individual differences in neophobic behavior might alternate each year: converging from winter to the breeding season, and then diverging again from the breeding season to winter.

Similarly, predictable alternation of convergence and divergence in individual differences might occur when animals are alternately exposed to high and low tides. Cornwell et al. (2019) studied individual differences using an assay of “boldness” in snails (*Littoraria irrorata*) under conditions simulating the tidal patterns in their natural habitat. For the same set of subjects, VARamg was higher at high tide than at low tide, and the value of CORR_i,s_ when the intercept was set at high tide was significantly negative, indicating convergence from high to low tide. There was also evidence that rank‐order consistency was largely maintained from high to low tide. Given that in the snails' world, high and low tides regularly alternate with one another, these data imply that boldness in this species might regularly converge from high to low tide, and then diverge again from low to high tide.

Also, while we focused on individual phenotypic differences in this article, the approaches and statistical models described herein could easily be modified to study temporal changes in genotypic differences in behavioral or physiological traits. A number of authors have conducted longitudinal studies of behavioral change for genotypes in the laboratory (e.g., Edenbrow & Croft, 2011; Laskowski et al., 2022; Stamps et al., 2018) or pedigreed individuals in the laboratory or field (e.g., Class et al., 2019; Dingemanse, Barber, et al., 2012; Ronald, 2011; White & Wilson, 2019). In such cases, time‐specific statistics comparable to those described in this article could be used to determine whether, and if so when, convergence and divergence occurred at the genetic level. For instance, a study of genotypic differences in aversive conditioning in larval *Drosophila melanogaster* showed that when genotypes which expressed significantly different mean values when naïve were exposed to a single aversive training experience, their scores converged to similar scores (Stamps et al., 2018). Similarly, a field study of pedigreed great tits (*Parus major*), reported that additive genetic variance in exploratory behavior declined from year 0 to year 3, and that rank‐order consistency at the genetic level was not maintained over this period, as the cross‐year correlation dropped to zero (Class et al., 2019).

In some cases, the patterns suggested by the methods described in this article should themselves be viewed as preliminary, and warrant more complicated statistical models and/or more extensive datasets to determine when temporal changes in individual differences occurred. A simple example is when theory or preliminary analyzes suggest that individual reaction norms might be curved rather than linear. In that case, one could include polynomial terms for time in a random regression model to capture this relationship. However, this procedure requires observations at multiple time points for every subject for a meaningful and precise analysis (e.g., see Bell & Peeke, 2012; Goold & Newberry, 2017).

A more complicated example is illustrated by the worked example for the stickleback data from Jolles et al. (2019) described in this article. Our analyzes suggested that in this dataset, a period of possible maintenance for the first 2 weeks was followed by a period of divergence, which continued until the end of the study. However, those results were based on a standard random regression model, which assumes that individual deviations from the mean are linear. As a result, this type of model would be unable to capture a situation in which a period of strict maintenance (VAR_slope_ = 0) was immediately followed by a period of strong divergence (VAR_slope_ ≫ 0), as shown in Figure 2b. In order to more firmly establish that an initial period of maintenance was followed by a period of divergence, it would be useful to conduct a new empirical study with more samples per subject, which could then be analyzed using statistical models which do not rely on this assumption. For instance, if multiple samples per subject were collected once a week using the burst design, discrete time (character state) models similar to those described in this article could be used to determine if and when the patterns changed. Conversely, if multiple samples within each period were collected using a continuous design, then one could use a more complicated version of the random regression model described here to address this question. For example, one could create a dummy variable that divides time into two intervals (weeks 1–3 vs. 4–6), and then introduce this factor variable into a random regression model, where this effect is crossed with continuous time variable at fixed and random effects levels, permitting different among‐subjects variances to be fit within each interval (see Singer & Willett, 2003). Thus, such a model could capture patterns of individual variance as depicted in Figure 2b. Such a model would have the following coding structure in *R*:

$$Y\sim 1+\text{interval}+\text{time}+{\text{interval}}^{*}\;\text{time}+\left(1+\text{interval}+\text{time}+{\text{interval}}^{*}\;\text{time}\;|\;\mathrm{ID}\right).$$

This code would generate unique intercept and slope predictions for each individual within each interval, and permit testing of whether a period of maintenance (weeks 1–3) was followed by a period of divergence (weeks 4–6). In addition, one could determine exactly when changes occurred by fitting models with different interval cutoff points (e.g., weeks 1–2 vs. 3–6 in the fish study), and finding which best fit the data. For an example of this sort of analysis, see (Biro, 2012).

We also note that although in this article we focused on individual differences in predicted mean values, individuals may also differ from one another with respect to other variables which are used to describe temporally labile behavioral or physiological traits. One example is rIIV, or residual intraindividual variability, which indicates the extent to which each individual's scores vary around its mean value. In recent years, several studies have demonstrated that individual differences in rIIV are repeatable (Biro & Adriaenssens, 2013; Cornwell et al., 2023; Highcock & Carter, 2014), and the repeatability of rIIV implies some level of rank‐order consistency in this variable over time. However, as we have seen in this article, repeatability can be high during periods when individual differences are either converging or diverging, as well as when they are being maintained. In addition, to date, nearly every empirical study which has demonstrated individual differences in rIIV has been based on estimates of each subject's variability over the entire study (Mitchell et al., 2021).

But the evidence that average levels of rIIV can change over time (see above) raises the obvious question of whether individual differences in rIIV might also change (converge or diverge) over time. As was the case for individual differences in mean values, theoreticians and empiricists have offered suggestions on why we might expect to observe changes in individual differences in rIIV over time. For instance, Bayesian models of development predict that if individuals initially differ with respect to the variability of their behavior, those differences will decline over time if every individual is repeatedly exposed to the same cues or experiences (Stamps & Krishnan, 2014a). However, at present, there is only indirect evidence that individual differences in rIIV might change over time. For instance, a comparison of two groups of athletes (trained vs. novices) revealed that the trained athletes initially had lower levels of trial‐to‐trial variability than novices when both begin to learn a novel throwing task, but eventually both groups converged on similarly low levels of trial‐to‐trial variability for that task (Cohen & Sternad, 2009). We suggest that studies of temporal changes in individual differences in rIIV might be a profitable topic for future research, and predict that in the near future, methods following from those described in this article will be developed to detect convergence or divergence patterns for individual differences in rIIV.

## AUTHOR CONTRIBUTIONS

**Judy A. Stamps:** Conceptualization (equal); data curation (equal); formal analysis (supporting); investigation (equal); methodology (supporting); writing – original draft (lead). **Peter A. Biro:** Conceptualization (equal); data curation (equal); formal analysis (lead); investigation (equal); methodology (lead); software (lead); writing – original draft (supporting).

## Supporting information


Appendix S1–S4

## Data Availability

No new data has been used in this manuscript.

## References

[ece310615-bib-0001] Ackerman, P. L. (2007). New developments in understanding skilled performance. Current Directions in Psychological Science, 16, 235–239. 10.1111/j.1467-8721.2007.00487.x PMC2826813 20182651

[ece310615-bib-0002] Anglim, J. , & Wynton, S. K. A. (2015). Hierarchical Bayesian models of subtask learning. Journal of Experimental Psychology‐Learning Memory and Cognition, 41, 957–974. 25603165 10.1037/xlm0000103

[ece310615-bib-0003] Avery, S. N. , & Blackford, J. U. (2016). Slow to warm up: The role of habituation in social fear. Social Cognitive and Affective Neuroscience, 11, 1832–1840. 27445209 10.1093/scan/nsw095 PMC5091686

[ece310615-bib-0004] Beckmann, C. , & Biro, P. A. (2013). On the validity of a single (boldness) assay in personality research. Ethology, 119, 937–947.

[ece310615-bib-0005] Beerse, M. , Bigelow, K. E. , & Barrios, J. A. (2020). The patterning of local variability during the acquisition of a novel whole‐body continuous motor skill in young adults. Experimental Brain Research, 238, 1797–1812. 32494848 10.1007/s00221-020-05840-9

[ece310615-bib-0006] Bell, A. M. , Hankison, S. J. , & Laskowski, K. L. (2009). The repeatability of behaviour: A meta‐analysis. Animal Behaviour, 77, 771–783. 24707058 10.1016/j.anbehav.2008.12.022 PMC3972767

[ece310615-bib-0007] Bell, A. M. , & Peeke, H. V. S. (2012). Individual variation in habituation: Behaviour over time toward different stimuli in threespine sticklebacks (*Gasterosteus aculeatus*). Behaviour, 149, 1339–1365. 25678715 10.1163/1568539X-00003019 PMC4323190

[ece310615-bib-0008] Bergmuller, R. , & Taborsky, M. (2010). Animal personality due to social niche specialisation. Trends in Ecology & Evolution, 25, 504–511. 20638151 10.1016/j.tree.2010.06.012

[ece310615-bib-0009] Beveridge, D. , Mitchell, D. J. , Beckmann, C. , & Biro, P. A. (2022). Weak evidence that asset protection underlies temporal or contextual consistency in boldness of a terrestrial crustacean. Behavioral Ecology and Sociobiology, 76, 94.

[ece310615-bib-0010] Bierbach, D. , Laskowski, K. L. , & Wolf, M. (2017). Behavioural individuality in clonal fish arises despite near‐identical rearing conditions. Nature Communications, 8, 15361. 10.1038/ncomms15361 PMC5442312 28513582

[ece310615-bib-0011] Biro, P. A. (2012). Do rapid assays predict repeatability in labile (behavioural) traits? Animal Behaviour, 83, 1295–1300.

[ece310615-bib-0012] Biro, P. A. , & Adriaenssens, B. (2013). Predictability as a personality trait: Consistent differences in intraindividual behavioral variation. American Naturalist, 182, 621–629. 10.1086/673213 24107369

[ece310615-bib-0013] Biro, P. A. , Adriaenssens, B. , & Sampson, P. (2014). Individual and sex‐specific differences in intrinsic growth rate covary with consistent individual differences in behaviour. Journal of Animal Ecology, 83, 1186–1195. 24673423 10.1111/1365-2656.12210

[ece310615-bib-0014] Biro, P. A. , & Stamps, J. A. (2015). Using repeatability to study physiological and behavioural traits: Ignore time‐related change at your peril. Animal Behaviour, 105, 223–230.

[ece310615-bib-0015] Brommer, J. E. (2013). Variation in plasticity of personality traits implies that the ranking of personality measures changes between environmental contexts: Calculating the cross‐environmental correlation. Behavioral Ecology and Sociobiology, 67, 1709–1718.

[ece310615-bib-0016] Brommer, J. E. , & Class, B. (2015). The importance of genotype‐by‐age interactions for the development of repeatable behavior and correlated behaviors over lifetime. Frontiers in Zoology, 12, S2. 26816518 10.1186/1742-9994-12-S1-S2 PMC4722339

[ece310615-bib-0017] Brust, V. , Schindler, P. M. , & Lewejohann, L. (2015). Lifetime development of behavioural phenotype in the house mouse (*Mus musculus*). Frontiers in Zoology, 12, S17. 26816516 10.1186/1742-9994-12-S1-S17 PMC4722345

[ece310615-bib-0018] Burki, C. N. , Ludwig, C. , Chicherio, C. , & De Ribaupierre, A. (2014). Individual differences in cognitive plasticity: An investigation of training curves in younger and older adults. Psychological Research‐Psychologische Forschung, 78, 821–835. 10.1007/s00426-014-0559-3 24652343

[ece310615-bib-0019] Burt, K. B. , & Obradovic, J. (2013). The construct of psychophysiological reactivity: Statistical and psychometric issues. Developmental Review, 33, 29–57.

[ece310615-bib-0020] Carlson, B. E. , & Tetzlaff, S. J. (2020). Long‐term behavioral repeatability in wild adult and captive juvenile turtles (*Terrapene carolina*): Implications for personality development. Ethology, 126, 668–678.

[ece310615-bib-0021] Carter, A. J. , Feeney, W. E. , Marshall, H. H. , Cowlishaw, G. , & Heinsohn, R. (2013). Animal personality: What are behavioural ecologists measuring? Biological Reviews, 88, 465–475. 23253069 10.1111/brv.12007

[ece310615-bib-0022] Caspi, A. , Roberts, B. W. , & Shiner, R. L. (2005). Personality development: Stability and change. Annual Review of Psychology, 56, 453–484. 10.1146/annurev.psych.55.090902.141913 15709943

[ece310615-bib-0023] Cavanagh, J. F. , Kumar, P. , Mueller, A. A. , Richardson, S. P. , & Mueen, A. (2018). Diminished EEG habituation to novel events effectively classifies Parkinson's patients. Clinical Neurophysiology, 129, 409–418. 29294412 10.1016/j.clinph.2017.11.023 PMC5999543

[ece310615-bib-0024] Class, B. , & Brommer, J. E. (2016). Senescence of personality in a wild bird. Behavioral Ecology and Sociobiology, 70, 733–744.

[ece310615-bib-0025] Class, B. , Brommer, J. E. , & Van Oers, K. (2019). Exploratory behavior undergoes genotype‐age interactions in a wild bird. Ecology and Evolution, 9, 8987–8994. 31462997 10.1002/ece3.5430 PMC6706179

[ece310615-bib-0026] Cleasby, I. R. , Nakagawa, S. , & Schielzeth, H. (2015). Quantifying the predictability of behaviour: Statistical approaches for the study of between‐individual variation in the within‐individual variance. Methods in Ecology and Evolution, 6, 27–37.

[ece310615-bib-0027] Cohen, J. (1988). Statistical power analysis for the behavioral sciences. Lawrence Erlbaum Associates.

[ece310615-bib-0028] Cohen, R. G. , & Sternad, D. (2009). Variability in motor learning: Relocating, channeling and reducing noise. Experimental Brain Research, 193, 69–83. 18953531 10.1007/s00221-008-1596-1 PMC2756422

[ece310615-bib-0029] Colombo, J. , & Mitchell, D. W. (2009). Infant visual habituation. Neurobiology of Learning and Memory, 92, 225–234. 18620070 10.1016/j.nlm.2008.06.002 PMC2758574

[ece310615-bib-0030] Cornwell, T. O. , Mccarthy, I. D. , Snyder, C. R. A. , & Biro, P. A. (2019). The influence of environmental gradients on individual behaviour: Individual plasticity is consistent across risk and temperature gradients. Journal of Animal Ecology, 88, 511–520. 30516829 10.1111/1365-2656.12935

[ece310615-bib-0031] Cornwell, T. O. , Mitchell, D. J. , Beckmann, C. , & Joynson, A. (2023). Multi‐level repeatability indicates selection may act on both personality and predictability, but neither are state‐dependent. Animal Behaviour, 195, 85–92.

[ece310615-bib-0032] Davis, J. M. , & Stamps, J. A. (2004). The effect of natal experience on habitat preferences. Trends in Ecology & Evolution, 19, 411–416. 16701298 10.1016/j.tree.2004.04.006

[ece310615-bib-0033] D'Hooge, R. , & De Deyn, P. P. (2001). Applications of the Morris water maze in the study of learning and memory. Brain Research Reviews, 36, 60–90. 11516773 10.1016/s0165-0173(01)00067-4

[ece310615-bib-0034] Dingemanse, N. J. , Barber, I. , Wright, J. , & Brommer, J. E. (2012). Quantitative genetics of behavioural reaction norms: Genetic correlations between personality and behavioural plasticity vary across stickleback populations. Journal of Evolutionary Biology, 25, 485–496. 22236352 10.1111/j.1420-9101.2011.02439.x

[ece310615-bib-0035] Dingemanse, N. J. , Bouwman, K. M. , van de Pol, M. , Van Overveld, T. , Patrick, S. C. , Matthysen, E. , & Quinn, J. L. (2012). Variation in personality and behavioural plasticity across four populations of the great tit *Parus major* . Journal of Animal Ecology, 81, 116–126. 21692798 10.1111/j.1365-2656.2011.01877.x

[ece310615-bib-0036] Dingemanse, N. J. , Kazem, A. J. N. , Réale, D. , & Wright, J. (2010). Behavioural reaction norms: Animal personality meets individual plasticity. Trends in Ecology & Evolution, 25, 81–89. 19748700 10.1016/j.tree.2009.07.013

[ece310615-bib-0037] Dochtermann, N. A. , & Royaute, R. (2019). The mean matters: Going beyond repeatability to interpret behavioural variation. Animal Behaviour, 153, 147–150.

[ece310615-bib-0038] Dougherty, L. R. , & Guillette, L. M. (2018). Linking personality and cognition: A meta‐analysis. Philosophical Transactions of the Royal Society of London. Series B, Biological Sciences, 373, 20170282. 30104427 10.1098/rstb.2017.0282 PMC6107561

[ece310615-bib-0039] Edelaar, P. , & Bolnick, D. I. (2019). Appreciating the multiple processes increasing individual or population fitness. Trends in Ecology & Evolution, 34, 435–446. 30850175 10.1016/j.tree.2019.02.001

[ece310615-bib-0040] Edenbrow, M. , & Croft, D. P. (2011). Behavioural types and life history strategies during ontogeny in the mangrove killifish, *Kryptolebias marmoratus* . Animal Behaviour, 82, 731–741.

[ece310615-bib-0041] Falconer, D. S. (1981). Introduction to quantitative genetics (2nd ed.). Longman.

[ece310615-bib-0042] Fanson, K. V. , & Biro, P. A. (2018). Meta‐analytic insights into factors influencing the repeatability of hormone levels in agricultural, ecological, and medical fields. American Journal of Physiology‐Regulatory, Integrative and Comparative Physiology, 316, R101–R109. 30427725 10.1152/ajpregu.00006.2018

[ece310615-bib-0043] Fatima, Z. , Kovacevic, N. , Misic, B. , & Mcintosh, A. R. (2016). Dynamic functional connectivity shapes individual differences in associative learning. Human Brain Mapping, 37, 3911–3928. 27353970 10.1002/hbm.23285 PMC6867365

[ece310615-bib-0044] Fawcett, T. W. , & Frankenhuis, W. E. (2015). Adaptive explanations for sensitive windows in development. Frontiers in Zoology, 12, S3. 26816521 10.1186/1742-9994-12-S1-S3 PMC4722342

[ece310615-bib-0045] Fisher, D. N. , Brachmann, M. , & Burant, J. B. (2018). Complex dynamics and the development of behavioural individuality. Animal Behaviour, 138, E1–E6.

[ece310615-bib-0046] Fleeson, W. (2001). Toward a structure‐ and process‐integrated view of personality: Traits as density distributions of states. Journal of Personality and Social Psychology, 80, 1011–1027. 11414368

[ece310615-bib-0047] Foilb, A. R. , Lui, P. , & Romeo, R. D. (2011). The transformation of hormonal stress responses throughout puberty and adolescence. Journal of Endocrinology, 210, 391–398. 21746793 10.1530/JOE-11-0206

[ece310615-bib-0048] Fokkema, R. W. , Korsten, P. , Schmoll, T. , & Wilson, A. J. (2021). Social competition as a driver of phenotype‐environment correlations: Implications for ecology and evolution. Biological Reviews, 96, 2561–2572. 34145714 10.1111/brv.12768 PMC9290562

[ece310615-bib-0049] Franklin, K. A. , Nicoll, M. A. C. , Butler, S. J. , Norris, K. , Ratcliffe, N. , Nakagawa, S. , & Gill, J. A. (2022). Individual repeatability of avian migration phenology: A systematic review and meta‐analysis. Journal of Animal Ecology, 91, 1416–1430. 35385132 10.1111/1365-2656.13697 PMC9546039

[ece310615-bib-0050] Franks, V. R. , Ewen, J. G. , Mccready, M. , & Thorogood, R. (2020). Foraging behaviour alters with social environment in a juvenile songbird. Proceedings of the Royal Society. B, Biological Sciences, 287, 20201878. 10.1098/rspb.2020.1878 PMC7739504 33234077

[ece310615-bib-0051] Freund, J. , Brandmaier, A. M. , Lewejohann, L. , Kirste, I. , Kritzler, M. , Kruger, A. , Sachser, N. , Lindenberger, U. , & Kempermann, G. (2013). Emergence of individuality in genetically identical mice. Science, 340, 756–759. 23661762 10.1126/science.1235294

[ece310615-bib-0052] Freund, J. , Brandmaier, A. M. , Lewejohann, L. , Kirste, I. , Kritzler, M. , Kruger, A. , Sachser, N. , Lindenberger, U. , & Kempermann, G. (2015). Association between exploratory activity ad social individuality in genetically identical mice living in the same enriched environment. Neuroscience, 309, 140–152. 25987202 10.1016/j.neuroscience.2015.05.027

[ece310615-bib-0053] Geary, D. C. , Bailey, D. H. , Littlefield, A. , Wood, P. , Hoard, M. K. , & Nugent, L. (2009). First‐grade predictors of mathematical learning disability: A latent class trajectory analysis. Cognitive Development, 24, 411–429. 10.1016/j.cogdev.2009.10.001 PMC2813681 20046817

[ece310615-bib-0054] Goold, C. , & Newberry, R. C. (2017). Modelling personality, plasticity and predictability in shelter dogs. Royal Society Open Science, 4, 170618. 28989764 10.1098/rsos.170618 PMC5627104

[ece310615-bib-0055] Highcock, L. , & Carter, A. J. (2014). Intraindividual variability of boldness is repeatable across contexts in a wild lizard. PLoS ONE, 9, e95179. 24733271 10.1371/journal.pone.0095179 PMC3986370

[ece310615-bib-0056] Horth, L. (2003). Melanic body colour and aggressive mating behaviour are correlated traits in male mosquitofish (*Gambusia hotbrooki*). Proceedings of the Royal Society. B, Biological Sciences, 270, 1033–1040. 10.1098/rspb.2003.2348 PMC1691335 12803892

[ece310615-bib-0057] Howard, R. W. (2009). Individual differences in expertise development over decades in a complex intellectual domain. Memory & Cognition, 37, 194–209. 19223569 10.3758/MC.37.2.194

[ece310615-bib-0058] Jolles, J. W. , Briggs, H. D. , Araya‐Ajoy, Y. G. , & Boogert, N. J. (2019). Personality, plasticity and predictability in sticklebacks: Bold fish are less plastic and more predictable than shy fish. Animal Behaviour, 154, 193–202.

[ece310615-bib-0059] Kluen, E. , & Brommer, J. E. (2013). Context‐specific repeatability of personality traits in a wild bird: A reaction‐norm perspective. Behavioral Ecology, 24, 650–658.

[ece310615-bib-0060] Knοrnschild, M. , Nagy, M. , Metz, M. , Mayer, F. , & Von Helversen, O. (2012). Learned vocal group signatures in the polygynous bat *Saccopteryx bilineata* . Animal Behaviour, 84, 761–769.

[ece310615-bib-0061] Kok, E. M. A. , Burant, J. B. , Dekinga, A. , Manche, P. , Saintonge, D. , Piersma, T. , & Mathot, K. J. (2019). Within‐individual canalization contributes to age‐related increases in trait repeatability: A longitudinal experiment in red knots. American Naturalist, 194, 455–469. 10.1086/704593 31490730

[ece310615-bib-0062] Kraft, B. , Lemakos, V. A. , Travis, J. , & Hughes, K. A. (2018). Pervasive indirect genetic effects on behavioral development in polymorphic eastern mosquitofish. Behavioral Ecology, 29, 289–300.

[ece310615-bib-0063] Kraft, B. , Williams, E. , Lemakos, V. A. , Travis, J. , & Hughes, K. A. (2016). Genetic color morphs in the eastern mosquitofish experience different social environments in the wild and laboratory. Ethology, 122, 869–880.

[ece310615-bib-0064] Krakauer, J. W. , Hadjiosif, A. M. , Xu, J. , Wong, A. L. , & Haith, A. M. (2019). Motor learning. Comprehensive Physiology, 9, 613–663. 30873583 10.1002/cphy.c170043

[ece310615-bib-0065] Langley, E. J. G. , Van Horik, J. O. , Whiteside, M. A. , & Madden, J. R. (2018). Individuals in larger groups are more successful on spatial discrimination tasks. Animal Behaviour, 142, 87–93. 30147111 10.1016/j.anbehav.2018.05.020 PMC6107781

[ece310615-bib-0066] Laskowski, K. L. , Bierbach, D. , Jolles, J. W. , Doran, C. , & Wolf, M. (2022). The emergence and development of behavioral individuality in clonal fish. Nature Communications, 13, 6419. 10.1038/s41467-022-34113-y PMC9616841 36307437

[ece310615-bib-0067] Leussis, M. P. , & Bolivar, V. J. (2006). Habituation in rodents: A review of behavior, neurobiology, and genetics. Neuroscience and Biobehavioral Reviews, 30, 1045–1064. 16774787 10.1016/j.neubiorev.2006.03.006

[ece310615-bib-0068] Lohman, D. F. (1999). Minding our P's and Q's. In P. L. Ackerman , P. C. Kyllonen , & R. D. Roberts (Eds.), Learning and individual differences: Process, trait, and content determinants (pp. 55–76). American Psychological Association.

[ece310615-bib-0069] Luttbeg, B. , & Sih, A. (2010). Risk, resources and state‐dependent adaptive behavioural syndromes. Philosophical Transactions of the Royal Society of London. Series B, Biological Sciences, 365, 3977–3990. 21078650 10.1098/rstb.2010.0207 PMC2992746

[ece310615-bib-0070] Martin, J. G. A. , Nussey, D. H. , Wilson, A. J. , & Réale, D. (2011). Measuring individual differences in reaction norms in field and experimental studies: A power analysis of random regression models. Methods in Ecology and Evolution, 4, 362–374.

[ece310615-bib-0071] Martin, J. G. A. , & Reale, D. (2008). Temperament, risk assessment and habituation to novelty in eastern chipmunks, *Tamias striatus* . Animal Behaviour, 75, 309–318.

[ece310615-bib-0072] Mathot, K. J. , & Dall, S. R. X. (2013). Metabolic rates can drive individual differences in information and insurance use under the risk of starvation. American Naturalist, 182, 611–620. 10.1086/673300 24107368

[ece310615-bib-0073] Mathot, K. J. , Wright, J. , Kempenaers, B. , & Dingemanse, N. J. (2012). Adaptive strategies for managing uncertainty may explain personality‐related differences in behavioural plasticity. Oikos, 121, 1009–1020.

[ece310615-bib-0074] Mitchell, D. J. , Beckmann, C. , & Biro, P. A. (2021). Understanding the unexplained: The magnitude and correlates of individual differences in residual variance. Ecology and Evolution, 11, 7201–7210. 34188806 10.1002/ece3.7603 PMC8216950

[ece310615-bib-0075] Mitchell, D. J. , & Biro, P. A. (2017). Is behavioural plasticity consistent across different environmental gradients and through time? Proceedings of the Royal Society. B, Biological sciences, 284, 20170893. 10.1098/rspb.2017.0893 PMC5563805 28794220

[ece310615-bib-0076] Mitchell, D. J. , Fanson, B. G. , Beckmann, C. , & Biro, P. A. (2016). Towards powerful experimental and statistical approaches to study intraindividual variability in labile traits. Royal Society Open Science, 3, 160352. 27853550 10.1098/rsos.160352 PMC5098975

[ece310615-bib-0077] Mitchell, D. J. , & Houslay, T. M. (2021). Context‐dependent trait covariances: How plasticity shapes behavioral syndromes. Behavioral Ecology, 32, 25–29. 33708005 10.1093/beheco/araa115 PMC7937033

[ece310615-bib-0078] Montiglio, P. O. , Ferrari, C. , & Reale, D. (2013). Social niche specialization under constraints: Personality, social interactions and environmental heterogeneity. Philosophical Transactions of the Royal Society of London. Series B, Biological Sciences, 368, 20120343. 23569291 10.1098/rstb.2012.0343 PMC3638446

[ece310615-bib-0079] Moore, A. J. , Brodie, E. D. , & Wolf, J. B. (1997). Interacting phenotypes and the evolutionary process. 1. Direct and indirect genetic effects of social interactions. Evolution, 51, 1352–1362. 28568644 10.1111/j.1558-5646.1997.tb01458.x

[ece310615-bib-0080] Mottus, R. , Allik, J. , Hrebickova, M. , Koots‐Ausmees, L. , & Realo, A. (2016). Age differences in the variance of personality characteristics. European Journal of Personality, 30, 4–11.

[ece310615-bib-0081] Mottus, R. , Briley, D. A. , Zheng, A. Q. , Mann, F. D. , Engelhardt, L. E. , Tackett, J. L. , Harden, K. P. , & Tucker‐Drob, E. M. (2019). Kids becoming less alike: A behavioral genetic analysis of developmental increases in personality variance from childhood to adolescence. Journal of Personality and Social Psychology, 117, 635–658. 30920282 10.1037/pspp0000194 PMC6687565

[ece310615-bib-0082] Mottus, R. , Soto, C. J. , & Slobodskaya, H. R. (2017). Are all kids alike? The magnitude of individual differences in personality characteristics tends to increase from early childhood to early adolescence. European Journal of Personality, 31, 313–328.

[ece310615-bib-0083] Nakagawa, S. , & Schielzeth, H. (2010). Repeatability for Gaussian and non‐Gaussian data: A practical guide for biologists. Biological Reviews, 85, 935–956. 20569253 10.1111/j.1469-185X.2010.00141.x

[ece310615-bib-0084] Nespolo, R. F. , & Franco, M. (2007). Whole‐animal metabolic rate is a repeatable trait: A meta‐analysis. Journal of Experimental Biology, 210, 2000–2005. 17515425 10.1242/jeb.02780

[ece310615-bib-0085] Nesselroade, J. R. (1991). The warp and woof of the developmental fabric. In R. Downs , L. Liben , & D. Palermo (Eds.), Visions of development, the environment, and aesthetics: The legacy of Joachim F. Wohlwill (pp. 213–240). Erlbaum.

[ece310615-bib-0086] Ogorman, J. G. (1977). Individual differences in habituation of human physiological responses – Review of theory, method, and findings in study of personality correlates in non‐clinical populations. Biological Psychology, 5, 257–318. 338041 10.1016/0301-0511(77)90017-5

[ece310615-bib-0087] Oudman, T. , Bijleveld, A. I. , Kavelaars, M. M. , Dekinga, A. , Cluderay, J. , Piersma, T. , & van Gils, J. A. (2016). Diet preferences as the cause of individual differences rather than the consequence. Journal of Animal Ecology, 85, 1378–1388. 27306138 10.1111/1365-2656.12549

[ece310615-bib-0088] Perkeybile, A. M. , & Bales, K. L. (2017). Intergenerational transmission of sociality: The role of parents in shaping social behavior in monogamous and non‐monogamous species. Journal of Experimental Biology, 220, 114–123. 28057834 10.1242/jeb.142182 PMC5278619

[ece310615-bib-0089] Petelle, M. B. , Mccoy, D. E. , Alejandro, V. , Martin, J. G. A. , & Blumstein, D. T. (2013). Development of boldness and docility in yellow‐bellied marmots. Animal Behaviour, 86, 1147–1154.

[ece310615-bib-0090] Pigliucci, M. (2001). Phenotypic plasticity: Beyond nature and nurture. John Hopkins University Press.

[ece310615-bib-0091] Plomin, R. , Defries, J. C. , Knopik, V. S. , & Neiderhiser, J. M. (2016). Top 10 replicated findings from behavioral genetics. Perspectives on Psychological Science, 11, 3–23. 26817721 10.1177/1745691615617439 PMC4739500

[ece310615-bib-0092] Plomin, R. , Defries, J. C. , & Loehlin, J. C. (1977). Genotype‐environment interaction and correlation in analysis of human behavior. Psychological Bulletin, 84, 309–322. 557211

[ece310615-bib-0093] Polverino, G. , Cigliano, C. , Nakayama, S. , & Mehner, T. (2016). Emergence and development of personality over the ontogeny of fish in absence of environmental stress factors. Behavioral Ecology and Sociobiology, 70, 2027–2037.

[ece310615-bib-0094] Polverino, G. , Palmas, B. M. , Evans, J. P. , & Gasparini, C. (2019). Individual plasticity in alternative reproductive tactics declines with social experience in male guppies. Animal Behaviour, 148, 113–121.

[ece310615-bib-0095] Ramakers, J. J. C. , Visser, M. E. , & Gienapp, P. (2020). Quantifying individual variation in reaction norms: Mind the residual. Journal of Evolutionary Biology, 33, 352–365. 31746497 10.1111/jeb.13571 PMC7079083

[ece310615-bib-0096] Rast, P. , & Zimprich, D. (2009). Individual differences and reliability of paired associates learning in younger and older adults. Psychology and Aging, 24, 1001–1006. 20025414 10.1037/a0016138

[ece310615-bib-0097] Rescorla, R. A. , & Wagner, A. R. (1972). A theory of Pavlovian conditioning: Variations in the effectiveness of reinforcement and nonreinforcement. In A. H. Black & W. F. Prokasy (Eds.), Classical conditioning II: Current research and theory (pp. 64–99). Appleton‐Century‐Crofts.

[ece310615-bib-0098] Roberts, B. W. , & DelVecchio, W. F. (2000). The rank‐order consistency of personality traits from childhood to old age: A quantitative review of longitudinal studies. Psychological Bulletin, 126, 3–25. 10668348 10.1037/0033-2909.126.1.3

[ece310615-bib-0099] Rogosa, D. R. , & Willett, J. B. (1985). Understanding correlates of change by modeling individual differences in growth. Psychometrika, 50, 203–228.

[ece310615-bib-0100] Ronald, A. (2011). Is the child father of the Man'? Evaluating the stability of genetic influences across development. Developmental Science, 14, 1471–1478. 22010904 10.1111/j.1467-7687.2011.01114.x

[ece310615-bib-0101] Sakai, O. (2018). Comparison of personality between juveniles and adults in clonal gecko species. Journal of Ethology, 36, 221–228.

[ece310615-bib-0102] Sakai, O. (2020). Do different food amounts gradually promote personality variation throughout the life stage in a clonal gecko species? Animal Behaviour, 162, 47–56.

[ece310615-bib-0103] Salthouse, T. A. , & Nesselroade, J. R. (2010). Dealing with short‐term fluctuation in longitudinal research. The Journals of Gerontology. Series B, Psychological Sciences and Social Sciences, 65, 698–705. 20732931 10.1093/geronb/gbq060 PMC2954329

[ece310615-bib-0104] Saltz, J. B. , & Nuzhdin, S. V. (2014). Genetic variation in niche construction: Implications for development and evolutionary genetics. Trends in Ecology & Evolution, 29, 8–14. 24126050 10.1016/j.tree.2013.09.011 PMC3874263

[ece310615-bib-0105] Scarr, S. , & McCartney, K. (1983). How people make their own environments – A theory of genotype‐environment effects. Child Development, 54, 424–435. 6683622 10.1111/j.1467-8624.1983.tb03884.x

[ece310615-bib-0106] Shettleworth, S. (2010). Cognition, evolution and behavior. Oxford University Press.

[ece310615-bib-0107] Shmuelof, L. , Krakauer, J. W. , & Mazzoni, P. (2012). How is a motor skill learned? Change and invariance at the levels of task success and trajectory control. Journal of Neurophysiology, 108, 578–594. 22514286 10.1152/jn.00856.2011 PMC3404800

[ece310615-bib-0108] Sih, A. , Mathot, K. J. , Moiron, M. , Montiglio, P. O. , Wolf, M. , & Dingemanse, N. J. (2015). Animal personality and state‐behaviour feedbacks: A review and guide for empiricists. Trends in Ecology & Evolution, 30, 50–60. 25498413 10.1016/j.tree.2014.11.004

[ece310615-bib-0109] Singer, J. D. , & Willett, J. B. (2003). Applied longitudinal data analysis: Modeling change and event occurrence. Oxford University Press.

[ece310615-bib-0110] Stamps, J. A. , & Biro, P. A. (2016). Personality and individual differences in plasticity. Current Opinion in Behavioral Sciences, 12, 18–23.

[ece310615-bib-0111] Stamps, J. A. , Biro, P. A. , Mitchell, D. J. , & Saltz, J. B. (2018). Bayesian updating during development predicts genotypic differences in plasticity. Evolution, 72, 2167–2180. 30133698 10.1111/evo.13585

[ece310615-bib-0112] Stamps, J. A. , Briffa, M. , & Biro, P. A. (2012). Unpredictable animals: Individual differences in intraindividual variability (IIV). Animal Behaviour, 83, 1325–1334.

[ece310615-bib-0113] Stamps, J. A. , & Frankenhuis, W. E. (2016). Bayesian models of development. Trends in Ecology & Evolution, 31, 260–268. 26896042 10.1016/j.tree.2016.01.012

[ece310615-bib-0114] Stamps, J. A. , & Groothuis, T. G. G. (2010). The development of animal personality: Relevance, concepts and perspectives. Biological Reviews, 85, 301–325. 19961473 10.1111/j.1469-185X.2009.00103.x

[ece310615-bib-0115] Stamps, J. A. , & Krishnan, V. V. (2014a). Combining information from ancestors and personal experiences to predict individual differences in developmental trajectories. American Naturalist, 184, 647–657. 10.1086/678116 25325748

[ece310615-bib-0116] Stamps, J. A. , & Krishnan, V. V. (2014b). Individual differences in the potential and realized developmental plasticity of personality traits. Frontiers in Ecology and Evolution, 2, 69.

[ece310615-bib-0117] Stamps, J. A. , & Luttbeg, B. (2022). Sensitive period diversity: Insights from evolutionary models. Quarterly Review of Biology, 97, 243–295.

[ece310615-bib-0118] Stanovich, K. E. (1986). Mathew effects in reading – Some consequences of individual differences in the acquisition of literacy. Reading Research Quarterly, 21, 360–407.

[ece310615-bib-0119] Sternad, D. (2018). It's not (only) the mean that matters: Variability, noise and exploration in skill learning. Current Opinion in Behavioral Sciences, 20, 183–195. 30035207 10.1016/j.cobeha.2018.01.004 PMC6051545

[ece310615-bib-0120] Taff, C. C. , Schoenle, L. A. , & Vitousek, M. N. (2018). The repeatability of glucocorticoids: A review and meta‐analysis. General and Comparative Endocrinology, 260, 136–145. 29355531 10.1016/j.ygcen.2018.01.011

[ece310615-bib-0121] Tarantola, T. , Kumaran, D. , Dayan, P. , & De Martino, B. (2017). Prior preferences beneficially influence social and non‐social learning. Nature Communications, 8, 817. 10.1038/s41467-017-00826-8 PMC5635122 29018195

[ece310615-bib-0122] Thys, B. , Pinxten, R. , & Eens, M. (2021). Long‐term repeatability and age‐related plasticity of female behaviour in a free‐living passerine. Animal Behaviour, 172, 45–54.

[ece310615-bib-0123] Torquet, N. , Marti, F. , Campart, C. , Tolu, S. , Nguyen, C. , Oberto, V. , Benallaoua, M. , Naude, J. , Didienne, S. , Debray, N. , Jezequel, S. , Le Gouestre, L. , Hannesse, B. , Mariani, J. , Mourot, A. , & Faure, P. (2018). Social interactions impact on the dopaminergic system and drive individuality. Nature Communications, 9, 3081. 10.1038/s41467-018-05526-5 PMC6079008 30082725

[ece310615-bib-0124] Trappes, R. , Nematipour, B. , Kaiser, M. I. , Krohs, U. , Van Benthem, K. J. , Ernst, U. R. , Gadau, J. , Korsten, P. , Kurtz, J. , Schielzeth, H. , Schmoll, T. , & Takola, E. (2022). How individualized niches arise: Defining mechanisms of niche construction, niche choice, and niche conformance. Bioscience, 72, 538–548. 35677293 10.1093/biosci/biac023 PMC9169896

[ece310615-bib-0125] Trimmer, P. C. , Mcnamara, J. M. , Houston, A. I. , & Marshall, J. A. R. (2012). Does natural selection favour the Rescorla‐Wagner rule? Journal of Theoretical Biology, 302, 39–52. 22619751 10.1016/j.jtbi.2012.02.014

[ece310615-bib-0126] Urszan, T. J. , Garamszegi, L. Z. , Nagy, G. , Hettyey, A. , Torok, J. , & Herczeg, G. (2018). Experience during development triggers between‐individual variation in behavioural plasticity. Journal of Animal Ecology, 87, 1264–1273. 29752882 10.1111/1365-2656.12847

[ece310615-bib-0127] van de Pol, M. (2012). Quantifying individual variation in reaction norms: How study design affects the accuracy, precision and power of random regression models. Methods in Ecology and Evolution, 3, 268–280.

[ece310615-bib-0128] Ventura, A. K. , & Worobey, J. (2013). Early influences on the development of food preferences. Current Biology, 23, R401–R408. 23660363 10.1016/j.cub.2013.02.037

[ece310615-bib-0129] West‐Eberhard, M. J. (2003). Developmental plasticity and evolution. Oxford University Press.

[ece310615-bib-0130] Westneat, D. F. , Wright, J. , & Dingemanse, N. J. (2015). The biology hidden inside residual within‐individual phenotypic variation. Biological Reviews, 90, 729–743. 25080034 10.1111/brv.12131

[ece310615-bib-0131] White, C. R. , Schimpf, N. G. , & Cassey, P. (2013). The repeatability of metabolic rate declines with time. Journal of Experimental Biology, 216, 1763–1765. 23264481 10.1242/jeb.076562

[ece310615-bib-0132] White, S. J. , & Wilson, A. J. (2019). Evolutionary genetics of personality in the Trinidadian guppy I: Maternal and additive genetic effects across ontogeny. Heredity, 122, 1–14. 29773896 10.1038/s41437-018-0082-1 PMC6288082

[ece310615-bib-0133] Wilder, J. (1931). The “excretion value principle”, an unobserved biological law and its significances for research and practice. Zeitschrift Fur Die Gesamte Neurologie Und Psychiatrie, 137, 317–338.

[ece310615-bib-0134] Wilder, J. (1965). Pitfalls in the methodology of the law of initial value. American Journal of Psychotherapy, 19, 577–584. 5837158 10.1176/appi.psychotherapy.1965.19.4.577

[ece310615-bib-0135] Wilson, A. D. M. , & McLaughlin, R. L. (2007). Behavioural syndromes in brook charr, *Salvelinus fontinalis*: Prey‐search in the field corresponds with space use in novel laboratory situations. Animal Behaviour, 74, 689–698.

[ece310615-bib-0136] Wolak, M. E. , Fairbairn, D. J. , & Paulsen, Y. R. (2012). Guidelines for estimating repeatability. Methods in Ecology and Evolution, 3, 129–137.

[ece310615-bib-0137] Zuur, A. , Ieno, E. N. , Walker, N. , Saveliev, A. A. , & Smith, G. M. (2009). Mixed effects models and extensions in ecology with R. Springer.

