# Supplementary material for: Time‐specific convergence and divergence in individual differences in behavior: Theory, protocols and analyzes
Source: Ecol Evol. 2023 Nov 28;13(11):e10615. doi: 10.1002/ece3.10615 (PMC10682899; doi:10.1002/ece3.10615)
Supplement: Supplementary file 1 — Appendix S1–S4 [file ECE3-13-e10615-s001.docx]

**APPENDIX S1** Code in R for the worked example of the random regression model, using data from Jolles et al. (2019)

# This annotated code is made up of three major parts:

#

# A) reading in and visualising data, running initial quick analyses and fit checks

# B) running analysis in bayesian mcmc model that permits residual modelling and facilitates part 3

# C) estimating time specific a) variances b) among subjects correlations of means c) repeatability

#################################################

#### PART A explore data, initial analysis ####

#################################################

setwd("C:/Users/pbiro/Pete/Research/CONTINUING work/2020 temporal change w Judy")

ds<-read.csv("jolles.csv") # read in data set, call it "ds"

View(ds) # check data read in correctly, check variable names

#### restrict analysis to days 1-6, and remove day=1.15 ######

#### read Jolles for why we do this

dsx <- subset(ds, testwk<10 & testwk!=1.15 ) # subset data

View(dsx) # check subset worked (always check things)

### basic raw data plots as first step ###

library(ggplot2) # open plotting package

ds1 <- subset(dsx, id>40&id<53) # select subset of IDs to plot

ggplot(ds1, aes(x = testwk, y = prop.out, group = id)) + # plot raw data by ID

geom_point(size = 2) + # size of data points

facet_wrap(. ~ id) # produces one plot per ID

##### transform data to normalise residuals #####

hist(dsx$prop.out) # plot data: proportion bit skewed, will affect residuals

dsx$asinprop <- asin(sqrt(dsx$prop.out)) # arc-sine sqrt transform

hist(dsx$asinprop) # re-plot data: normal now

#### left-centre time so intercept has better meaning #######

dsx$test <- dsx$testwk - 1 # left-centre test week

#### preliminary analyses using lme4

#### fast to run, easy plots of model predictions

library(lme4) # open package

fit1<-lmer(asinprop ~ 1 + test + (1+test|id), # model structure

data=dsx, REML = TRUE)

summary(fit1) # generate model output

### how is model performing? #######

#### Plot predictions against raw data ###

library(broom) # open broom package to assist data wrangling

z<-augment(fit1) # get variables and fitted values from model

head(z) # view column names of "z" above

z2 <- subset(z, id>40&id<53 ) # select same subset of IDs to plot

library(ggplot2)

ggplot(z2, aes(x = test, y = asinprop, group = id)) + # same plot as above

geom_point(size = 2) +

geom_line(aes(y = .fitted), # adds lines for the fitted values

colour = 'red', alpha = 0.6) +

facet_wrap(. ~ id)

######## easy plot of all individual trends about the mean ###################

ggplot(z, aes(x = test, y = asinprop, group = id)) +

geom_line(aes(y = .fitted), # note this will look odd if there are additional fixed effects

colour = 'red',

alpha = 0.6)

##### check assumptions: plot all residual values against the fitted

ggplot(z, aes(x = .fitted, y = .resid)) + # same plot as before

geom_point(size = 2)

##### check assumptions: are intercepts and slopes normally distributed?

blups<-coef(fit1)$id

View(blups)

hist(blups$`(Intercept)`) # note slight skew in these, fixed in model in part 2

hist(blups$test) # slope predictions

#######################################

#### PART B Use bayesian model in brms ####

#######################################

##### now use bayesian model in brms ####

##### permits easy estimates of credible intervals (CIs), and residual modelling ####

library(brms)

lat<-brmsformula(asinprop ~ 1 + test + (1+test|id), sigma~0+factor(test)) # same model as above, but now residual model added

fit2 <- brm(lat, data=dsx,

prior = c(set_prior("normal(0,5)",class="b"), # priors are set here

set_prior("cauchy(0,2)",class="sd"),

set_prior("lkj(2)",class="cor")),

warmup = 500, iter = 2000, chains=4, cores=4, control=list(adapt_delta=0.97))

print(fit2, digits=3)

summary(fit2) # generate model output

# here can assess 'significance' of fitting residuals at each level of time

waic1 <- waic(fit2) # get WAIC value for model with categ time resid

waic0 <- waic(fit2) # re-run above model without "sigma" portion, then run this line

loo_compare(waic0, waic1) # generates WAIC value

####### plot temporal RNs and mean trend #########

######### from above brms parameters ##########

### this code provides temporal trendlines (RNs) after accounting for any fixed effects ..

### .. to isolate the temporal trendlines

pred <- as.data.frame(ranef(fit2)$id) # extract ID-specific deviations from mean (blups)

head(pred) # view the new data frame to identify column names

names(pred)[1] <- 'int' # rename column 1 to 'int' for intercept values

names(pred)[5] <- 'test' # rename col 5 to 'test' for slope values

hist(pred$int) #check to see if int's are normally distributed

hist(pred$test) #check to see if slopes are normally distributed

b<-fixef(fit2) # extract fixed effect parameters to 'b'

head(b) # view b, one row two columns

## range of values you want to plot over, given by testwk values

x <- 0:5

## un mute next two lines to create images for publication

# tiff(file="SuppFig2.tiff", width = 480, height = 480)

# pdf(file="Fig. 1.pdf", height=4, width=4)

plot(NULL, xlim=c(-1,6), ylim=c(0,1.2), axes=F, ylab="", xlab="")

mtext("Arcsine(Proportion)", side=2, line=2.5, cex=1.8)

mtext("Time (week)", side=1, line=2.5, cex=1.8)

axis(1, at = seq(-0,5,1), labels = c('1','2','3','4','5','6'))

axis(2, at = seq(0,1.2,0.1), labels=T)

for(j in 1:80){ # plots trend for each ID

lines(b[1] + pred$int[j] + (b[2]+pred$test[j])*x ~ x,

type = 'l', col = 'black', lwd = 1)

}

lines(b[1] +(b[2])*x ~ x, # adds mean trendline to plot

type = 'l', col = 'red', lwd = 2)

box()

dev.off()

###########################################################

#### PART C Calculate VARamgt, CORRt1,t2, CORRet,s, and Rt ####

###########################################################

### First we need to get predicted parameter values at each iteration

library(coda) # need coda package for as.mcmc

View(posterior_samples(fit2)) # view all predicted values of random effects

## random effects are fitted as sd's, not variance, so need to square them first

Vint <- (posterior_samples(fit2)$"sd_id__Intercept"^2) # get intercept sd and square

Vslope <- (posterior_samples(fit2)$"sd_id__test"^2) # get slope sd and square

corr <- (posterior_samples(fit2)$"cor_id__Intercept__test") # get COVi,s, expressed as correlation

Vresid0<- exp(posterior_samples(fit2)$"b_sigma_factortest0")^2 # residuals fit on log-link scale ...

Vresid1<- exp(posterior_samples(fit2)$"b_sigma_factortest1")^2 # so need to exp the values, then square

Vresid2<- exp(posterior_samples(fit2)$"b_sigma_factortest2")^2

Vresid3<- exp(posterior_samples(fit2)$"b_sigma_factortest3")^2

Vresid4<- exp(posterior_samples(fit2)$"b_sigma_factortest4")^2

Vresid5<- exp(posterior_samples(fit2)$"b_sigma_factortest5")^2

###########################

###### VARamgt ########

x<- 5 # enter time point here

COVis <- corr * sqrt(Vint) * sqrt(Vslope) # eqn for COVi,s from the corr

VARx <- Vint + 2*COVis*x + Vslope*(x^2) # VARamong for a given time x

mean(VARx);quantile(as.mcmc(VARx),probs = c(0.025, 0.975))

#######################

###### CORRt1,t2; #####

x<- 4 # specify lower time value of interval to consider

x1<- 5 # specify uppper time value

COVis <- corr * sqrt(Vint) * sqrt(Vslope) # eqn for COVi,s from the corr

VARx <- Vint + 2*COVis*x + Vslope*(x^2) # VARamong for a given time x

VARx1 <- Vint + 2*COVis*x1 + Vslope*(x1^2) # VARamong for a another time x1

COVxx1 <- Vint + COVis*x + COVis*x1 + Vslope*x*x1 # COV between two time points

corrxx1 <- COVxx1 / (sqrt(VARx)*sqrt(VARx1)) # corr between two time points

mean(corrxx1);quantile(as.mcmc(corrxx1),probs = c(0.025, 0.975))

##############################################

#### changing CORRet,s with changing x #######

x<- 5 # specify time value here

COVis <- corr * sqrt(Vint) * sqrt(Vslope) # calculate COVi,s from CORRi,s

COVx <- COVis + Vslope*x # calculate COVe,s at time t

VARx <- Vint + 2*COVis*x + Vslope*(x^2) # VARamg at time t

corris <- COVx / (sqrt(VARx) * sqrt(Vslope)) # re-express COVe,s to CORRe,s at time t

mean(corris);quantile(as.mcmc(corris),probs = c(0.025, 0.975))

################################################

### Repeatability (Rt) at each time point #######

x<- 1 # enter time value, from 0 to 5

COVis <- corr * sqrt(Vint) * sqrt(Vslope)

VARx <- Vint + 2*COVis*x + Vslope*(x^2)

#Rx <- VARx / (VARx + Vresid)

Rx <- VARx / (VARx + Vresid0) # enter same x value for resid

mean(Rx);quantile(as.mcmc(Rx),probs = c(0.025, 0.975))

mean(Vresid5);quantile(as.mcmc(Vresid5),probs = c(0.025, 0.975))

**APPENDIX S2.** Using random regression to analyze data collected using a continuous experimental design

A. A worked example, based on data from Jolles et al. (2019)(see text)

We begin by plotting each subject’s data over time, and inspecting the data for each subject to determine whether their temporal trajectories might be approximated by straight lines (Fig. A2.1; see Appendix S1 for the code used to generate this graph). In this case, this process suggested that the assumption of linearity might be reasonable.


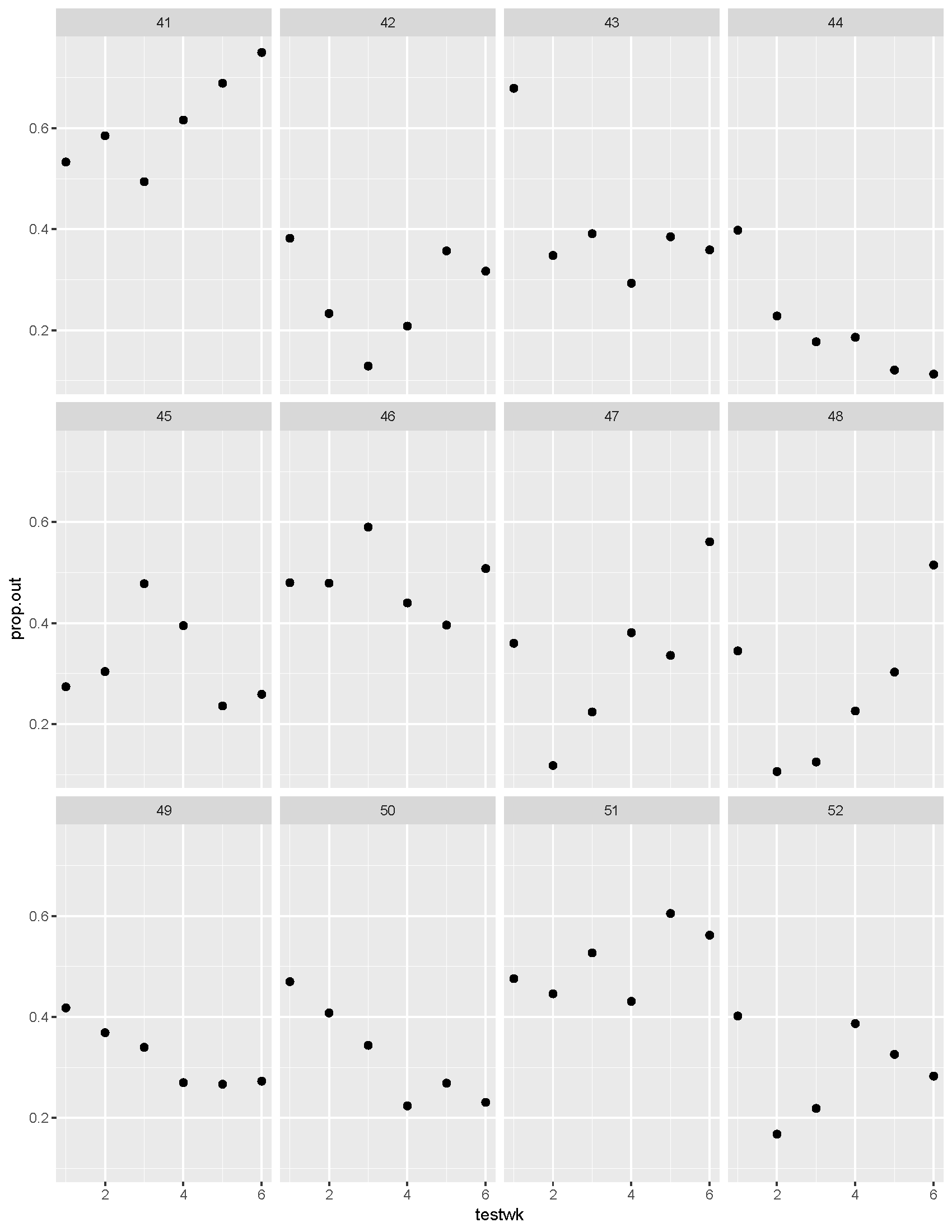


Figure A2.1 Plots of the data showing the proportion of time spent out of shelter each week, for an arbitrary selection of subjects (ID numbers 41 – 52) from the Jolles et al. (2019) data set.

Given the data was proportion data, bounded by zero and one, normality of residuals and constant variance were unlikely (indeed, the data had some left skew). Thus, we arcsine square root transformed the data prior to analyzes (see Appendix S1 for code). The linearity assumption can and should also be verified after the model is constructed using residual plots, or preferably, plots of the predicted reaction norms for each subject against the raw data for each subject (see Figure A2.2). Note also that random regression models assume that predicted intercepts and slopes (the ‘blups') are normally distributed (see below, S2B.2).

Next, we fit an initial exploratory random regression model to these transformed data using the widely used program lme4 in the statistical package R, using the following generalised notation:

Y ~ 1 + *time* + (1 + *time* | ID). (1)

The first instance of “1” in Eqn. 1 indicates a fixed effect intercept (usually not made explicit), and *time* indicates a fixed effect slope for time; together these two terms define the population level (mean) trendline across subjects. These same two terms given in parentheses specifies the among-subjects variance in their intercepts (predicted means at time zero; VAR_int_), and their predicted slopes across the study period (VAR_slope_) for the sample of subjects with unique identity numbers (labelled “ID”). Together, these two terms describe the extent to which the temporal trendlines of the subjects vary around the average trendline for the population; in addition, the covariance (specifically correlation) between intercepts and slopes (CORR_i,s_) is implicit in Eqn. 1 . When we fit this model (using lme4, model structure in Eqn 1, and the R code provided in Appendix S1) we obtained the output indicated in Table A2.1.

Table A2.1. Output from the exploratory random regression model of temporal change in the proportion of time spent out of shelter by sticklebacks (data from Jolles et al. (2019))

Random effects:

Groups Name Variance Std.Dev. Corr

id (Intercept) 0.013977 0.11823

test 0.001314 0.03625 -0.45

Residual 0.012851 0.11336

Number of obs: 470, groups: id, 80

Fixed effects:

Estimate Std. Error df t value Pr(>|t|)

(Intercept) 0.579880 0.016117 79.261531 35.980 < 2e-16 ***

test 0.016316 0.005106 78.580211 3.196 0.00201 **

In Table A2.1, the VARamg_t_ at the beginning of the study (at t = 0, which is week 1) is indicated by the random effects intercept variance (= 0.0139; note that this effect is also presented as an SD value as well, indicated by 0.118 in the second column). The variance among the subjects in their slopes is indicated by the random effects 'test' variance ( = 0.0013). The correlation between the intercepts at the beginning of the study and the slopes (CORR_i,s_) is indicated by the random effects Corr (= -0.45). The fixed effect slope for ‘test’ week (*b*=0.0163) indicates that on average, individuals increased the proportion of time they spent out of shelter over the 6 weekly test periods. We can test for the significance VARslopes using a Likelihood ratio test at this stage (it is significant), but we report its value and CI below.

Next, we can see how well this model performs in terms of capturing the individual patterns in the data by plotting the fitted values for each subject (i.e., its deviations + the fixed effect prediction) against its raw data points (see Appendix S1 for code). When we do so, we can see that the exploratory model does a reasonable job of predicting the temporal reaction norms for the subjects, and that the assumptions of linearity are largely met (Fig. A2.2). Note that because the model is left centered, this plot shows the first observation as occurring at test = 0, not at week = 1.

One could further examine standard residual plot of residuals against the fitted values, which are not shown here (code in Appendix S1). One should also plot the predicted intercepts and slopes to verify their normality, which was approximately met in this exploratory analysis, and fully met in the final analysis in which the residuals were allowed to vary through time (see Appendix S2B.2 for these plots, and Appendix S1 for the code used to generate them)


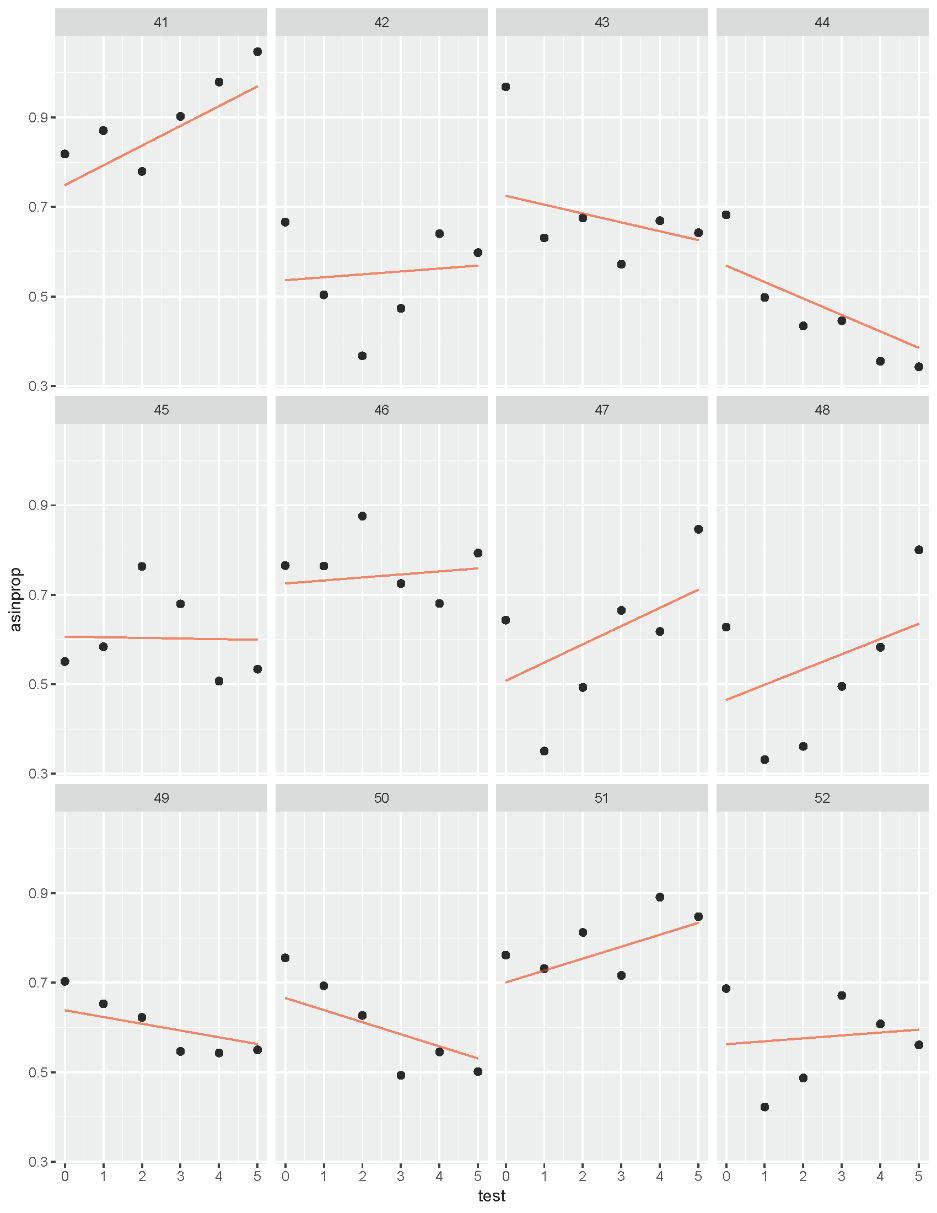


Figure A2.2. Plots of data showing the arcsine-square root transformed proportion of time spent out of shelter in relation to sampling week, for the same selection of individuals as in Fig A2.1. The lines fitted to each individual are the temporal reaction norms predicted from the exploratory random regression model. Note that as a result of the left centering in the random regression model, the independent variable, test, ranges from zero to five.

Finally, we can check to see what the exploratory model suggests about temporal changes in individual differences in the mean values of the subjects over the course of the study, by plotting the predicted trendlines for all of the subjects together in one plot (Fig. A2.3; see Appendix S1 for the code used to generate this figure).


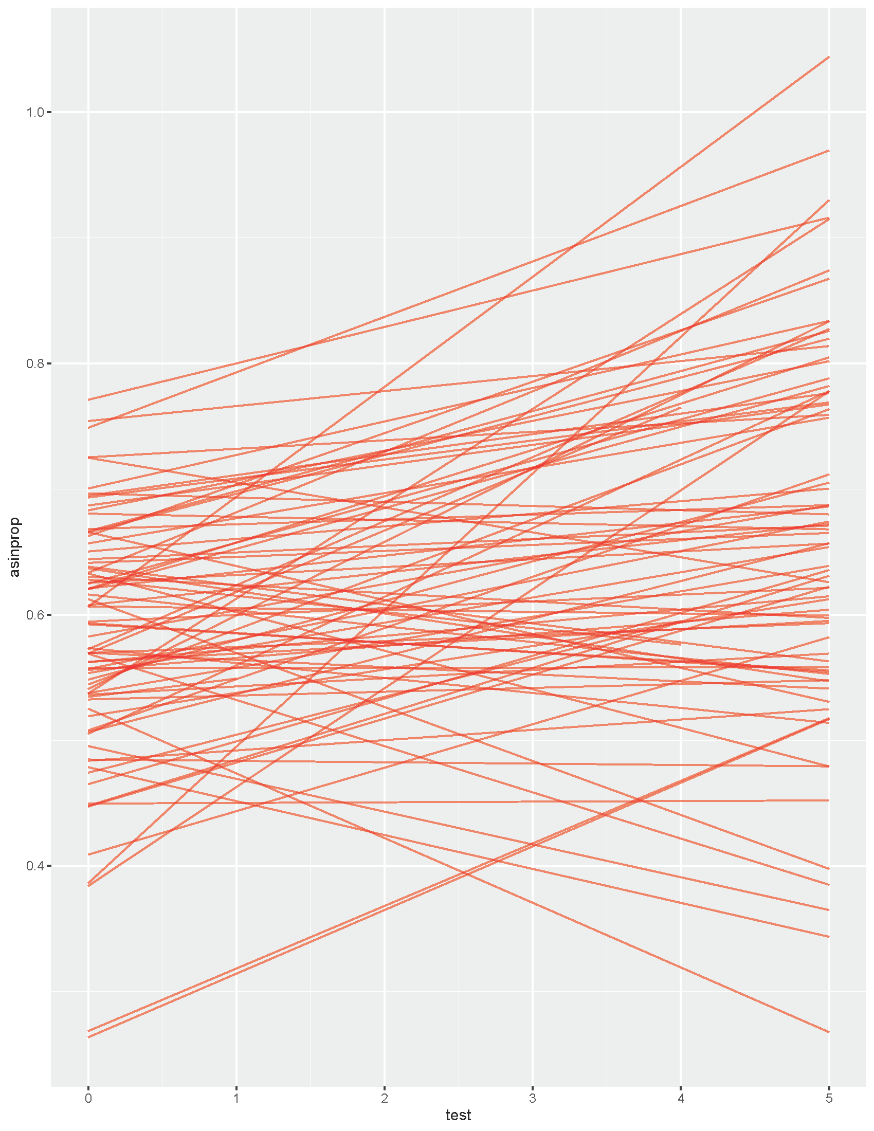


Figure A2.3. Fitted temporal reaction norms for 60 fish from exploratory random regression analysis of the data in Jolles et al. 2019.

The results generated by the exploratory model were ambiguous. The negative value of CORRi,s (see Table A2.1) might indicate convergence from week 1 to week 6, or it might indicate a situation in which an initial period of convergence was followed by a period of divergence (i.e., a reversal pattern, see Figure 3 in the text). The latter interpretation was supported by indications that the reaction norms of the subjects appeared to diverge later in the study (see Figure A2.3). In order to get a better idea about how individual differences changed over time, we must estimate the values of VARamg_t_, CORR_t1,t2_ and CORRe_t_,s at multiple weeks (t) over the course of the study.

To calculate these values, we refit the data using a different package in R: the Bayesian package brms (Bürkner, 2017). We used brms for two reasons. First, this program readily produces 95% credible intervals (CIs) for parameter estimates and other variables of interest calculated from them, such as VARamg_t_. It does so using posterior predicted values (parameter estimates predicted at each iteration of the model) which are then used to provide a probability distribution from which to estimate credible intervals. The latter are similar to confidence intervals, but are free of assumptions about the shape of the distributions. The second reason is that brms permits residual modelling, which allows us to assess if residual variation changes through time (whereas lme4 does not).

The coding syntax required for brms to define the model structure is the same as that already indicated in equation 1, but now we will also ask if residuals vary through time simply by adding a residual (or ‘dispersion’) model to the overall model to be fit:

sigma ~ 1 + factor(*time*) (2)

This notation for the residual SD (“sigma”) specifies the usual implicit single residual parameter (the overall mean, the intercept “1”) but now adds time, as a factor, to fit a residual standard deviation (SD) for each level of time (See Appendix S1 for full model statements). Fitting residuals separately by time for the Jolles data indicated sharp and significant declines in VARresid_t_ over the first three weeks (see Table A2.2), and the information criteria WAIC indicated that this effect was significant when compared to a model which assumed that residual variance was constant over time (see below). These results were supported by visual inspection of the plots for the individual fish, which indicated that for some of the subjects (e.g., 43, 47, 48 and 52 in Figure A2.2), variability around their trendlines was higher in weeks 1 and 2 than was the case later in the study.

Once the model was fitted, we extracted model predictions and generated the following plot (code provided in Appendix S1), which suggested a modest amount of crossing-over of the individual reaction norms in the first week or two, but mostly a pattern of divergence:


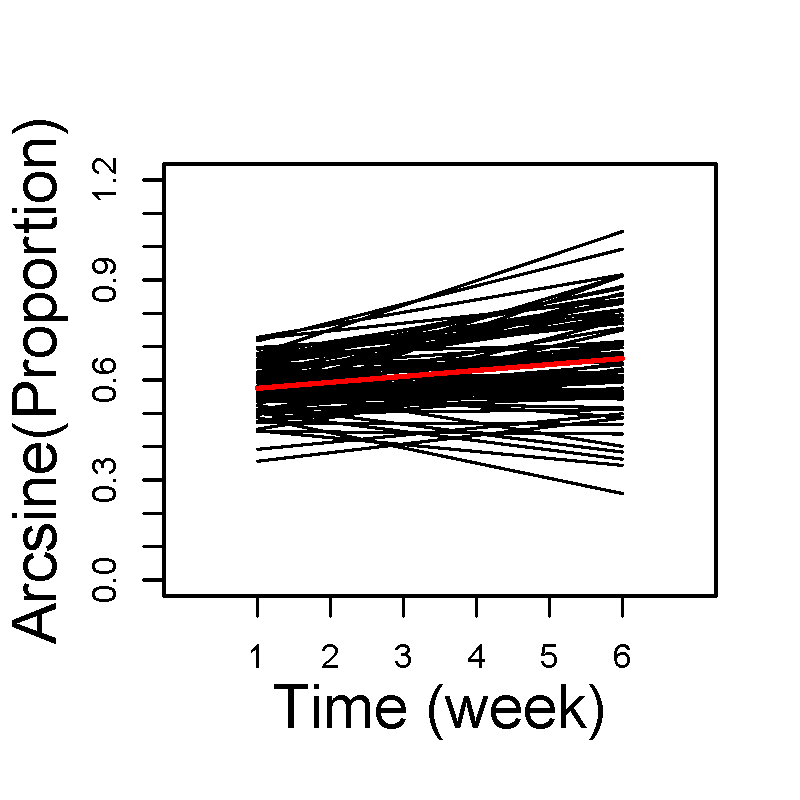


Figure A2.4. Predicted temporal reaction norms for the subjects in Jolles et al. (2019), generated by a random regression model that permits residual variance to vary over time. Black lines indicate the predicted reaction norms for each subject, and the red line indicates the mean level trend. Note that the predictions of this model differ from those of a model which assumes that the residuals do not vary over time (compare Figures A2.3 and A2.4). For a detailed comparison of the predictions of this model with those of a comparable model which assumes that residual variance is constant over time, see Section S2B, below.

We next used the estimates of VAR_int_, VAR_slope_, and the correlation between these (CORR_i,s_) generated by the brms model to calculate how five variables (VARamg_t_ , VARresid_t_, CORR_t1,t2_, CORRe_t_,s and repeatability R_t_) changed over the course of the study (results shown in Table A2.2).

VARamg_t_

The among subject’s variance can be estimated at any point in time (t) using the following standard equation:

VARamg_t_ = VAR_int_,+ 2*COV_i,s_ *t + VAR_slope_*t^2^ (3)

Because the program brms (and lme4) outputs CORR_i,s_ and not COV_i,s_, we need to convert the former to the latter, using the following equation:

COV_i,s_ = CORR_i,s_ * sqrt(VAR_int_) * sqrt(VAR_slope_) (4)

Then, we can use estimates of each of three parameters from each iteration of the model (the posteriors) and estimate this variance at any time point along with its credible interval, using the programming statements provided in Appendix S1.

Since the intercept is set at the beginning of the study (time = 0, week 1), VAR_int_ is the predicted VARamg_t_ at week 1. When we then vary the time (t) and compute VARamg_t_ for each week of the study using Eqn 3, our results suggest that VARamg_t_ was relatively low and very similar in weeks 1 and 2, and then it steadily increased from weeks 3 to 6 (Table A2.2, column A). The credible intervals (around these estimates indicate that VARamg_t_ was maintained at similar levels across the first two to three weeks of the study, but that it had increased thereafter and significantly diverged by the end of the study. For example, we can conclude the VARamg_t_ was significantly greater at week 6 than at weeks 1-3, based on the results showing that the CI at week 6 did not overlap with the CIs for weeks 1-3.

CORR_t1,t2_

We can estimate the among subjects’ correlation between predicted mean values between any two points in time, t=1 and t=2, using the standard equation:

CORR_t1,t2_ = COV_t1,t2_ / sqrt(VARamg_t1_)*sqrt(VARamg_t2_) (5)

The term COV_t1,t2_ is not provided by the estimated model parameters directly, and so we must use the following equation to obtain it:

COV_t1,t2_ = VAR_int_ + COV_i,s_*t1 + COV_i,s_*t2 + VAR_slope_*t2^2 (6)

The choice of values of t1 and t2 for estimates of CORR_t1,t2_ can vary among studies, e.g., as investigators focus on periods of special interest suggested by visual inspection of graphs of the predicted reaction norms. In the case of Jolles' data, CORR_t1,t2_ was relatively low early in the study (CORR_1,3_ = 0.82 [ CI = 0.67-0.93]), reflecting some crossing-over of the reaction norms that occurred over the period from week 1 to week 3 (see Figure A2.4). Later in the study, CORR_t1,t2_ was higher (CORR_4,6_ = 0.95 [ CI = 0.91-0.99]), reflecting the low levels of crossing-over that occurred after week 4. In order to determine the extent to which rank-order at the onset of a study was maintained during the remainder of the study, we suggest that investigators evaluate a series of values of CORR_t1,t2_ in which t1 is set at the beginning of the study and then t2 is set at successively later times during the study. When we did this for the Jolles data, we found that rank-order consistency was maintained throughout the study, although the strength of the correlation gradually diminished over time (Table A2.2, column C). For some research questions, it might even be useful to generate a matrix of CORR_t1,t2_ values for every pair of time periods in the study (e.g., Laskowski et al., 2022).

CORRe_t_,s

The correlation between the predicted means at different points in time (the subjects’ elevations at each time, e_t,_ ) and the slopes is given by:

CORRe_t_,s = COVe_t_, s/ (sqrt(VARamg_t_) * sqrt(VAR_slope_)) (7)

Estimating this correlation requires the COVe_t_,s, obtained by the following equation:

COVe_t_,s = COV_i,s_ + VAR_slope_*t (8)

In Jolles’ data set, CORRe_t_,s did not differ from zero in weeks 1 and 2, but was significantly positive from week 3 through week 6, indicating divergence from week 3 onwards (Table A2.2, column D).

R_t_ and VARresid_t_

When there is significant variance among the subjects in their slopes (VAR_slope_), one should use a slightly more complex formula to estimate R than is usually used for simple ‘conditional’ or unadjusted repeatability (Biro and Stamps, 2015). The general formulation for R is:

R = VARamg / VARamg + VARresid (9)

However, since VARamg can change over time, then R also needs to be estimated at specific points in time. This can be done by substituting Eqn 3 for the numerator in Eqn 9, in order to estimate VARamg_t_ at different points in time; VARresid_t_ is the estimated residual variance at each time, as we have fitted the residuals by time.

Analyzes of VARresid_t_ for the Jolles data indicated that the residual variance was higher in weeks 1 and 2 than it was later in the study (Table A2.2, column B). As a result of the increases in VARamg_t_ and decreases in VARresid_t_ over the course of the study period, R_t_ increased over time, and it was slightly higher by week 6 than it had been in weeks 1 or 2. Even so, the values of R_t_ were statistically significantly greater than zero throughout the study (Table A2.2, column E).

Table A2.2. Estimates for VARamg_t_, VARresid_t_, CORR_t1,t2_, CORRe_t_,s, and R_t_ presented for each time point derived from random regression analysis of the behavioral data from Jolles et al. (2019). Mean and CIs are indicated for each variable.

|  | A | B | C | D | E |
| --- | --- | --- | --- | --- | --- |
| Week | VARamg_t_ | VARresid_t_ | CORR_1,X_ | CORRe_t_,s | R_t_ |
| 1 | 0.0109 (0.005 – 0.019) | 0.028 (0.018 – 0.041) | NA | -0.18 (-0.52 – 0.38) | 0.28 (0.14-0.44) |
| 2 | 0.0105 (0.006 – 0.016) | 0.019 (0.013 – 0.027) | 0.95 (0.90-0.98) | 0.12 (-0.27 – 0.58) | 0.36 (0.22-0.51) |
| 3 | 0.0121 (0.008 – 0.018) | 0.009 (0.006 – 0.014) | 0.82 (0.67-0.93) | 0.39 (0.04 – 0.73) | 0.56 (0.42-0.70) |
| 4 | 0.0158 (0.01 – 0.023) | 0.009 (0.007 – 0.014) | 0.66 (0.42-0.87) | 0.59 (0.31 – 0.83) | 0.61 (0.48-0.73) |
| 5 | 0.0220 (0.015 – 0.031) | 0.008 (0.005 – 0.013) | 0.52 (0.21 – 0.81) | 0.72 (0.51 – 0.88) | 0.72 (0.59-0.84) |
| 6 | 0.0290 (0.020 – 0.043) | 0.011 (0.006 – 0.018) | 0.41 (0.07 – 0.76) | 0.81 (0.64 – 0.92) | 0.72 (0.58-0.85) |

B. A comparison of the results generated by random regression models that rely on different assumptions about changes in residual variance over time

Here, we use the data from Jolles et al. (2019) to compare the results generated by two random regression models, one of which allows for the possibility that VARresid might vary over time (the 'heterogenous residual' model, described above, in Section S2A), and an otherwise equivalent model that assumes that VARresid is constant over time (the 'constant residual' model). First, we show that the two models generate different estimates of variables required to describe and test for temporal changes in individual differences (VARamg_t_, R_t_, etc.). Second, we present graphs which demonstrate that the assumptions of normality of the random effects predictions are more closely met in the heterogeneous residual model than in the constant residual model.

1. Comparison of the results generated by the heterogeneous residual model and the constant residual model for the same data set

Table A2.3 shows the results generated by the heterogeneous residual model; this is the model described above, in Section S2A. In this model, we fit residual variation by week, by adding “sigma ~ 0 + factor(Week)” to our model statement when using the *brms* package in R.

Table A2.4 shows results from the constant residual model, which assumes that residual variation does not change over time (i.e., is constant). This is the type of random regression model that investigators often use to analyze temporal changes in individual differences.

First, allowing the residual variance to vary with time significantly improved model fit over the simpler model (delta WAIC = 22.2). The heterogeneous model not only indicates that VARresid_t_ did change over time, but also that those changes affected our estimates of other variables used to analyze temporal changes in individual differences. Note in particular how the significantly greater residual variance in weeks 1 and 2 in the heterogenous model than in the constant model (compare column B in Tables A2.3 and A2.4) generates different estimates of VARamg_t_, CORRe_t_,s and R_t_ for those weeks for the heterogeneous model than for the constant residual model (compare columns A, D and E in Tables A2.3 and A2.4). The greater residual variance in weeks 1 and 2 is also noticeable in plots of the lines fitted to the raw data (see Figure A2.2).

Table A2.3. Results for the heterogeneous residual variation model analysis of data from Jolles et al. (2019). This is the same table as Table A2.2 above, provided again here for easy comparison with Table A2.4, below. Estimates for VARamg_t_, VARresid_t_, CORR_t1,t2_, CORRe_t_,s, and R_t_ presented for each time point derived from random regression analysis. Mean and CIs are indicated for each variable.

|  | A | B | C | D | E |
| --- | --- | --- | --- | --- | --- |
| Week | VARamg_t_ | VARresid_t_ | CORR_1,X_ | CORRe_t_,s | R_t_ |
| 1 | 0.0109 (0.005 – 0.019) | 0.028 (0.018 – 0.041) | NA | -0.18 (-0.52 – 0.38) | 0.28 (0.14-0.44) |
| 2 | 0.0105 (0.006 – 0.016) | 0.019 (0.013 – 0.027) | 0.95 (0.90-0.98) | 0.12 (-0.27 – 0.58) | 0.36 (0.22-0.51) |
| 3 | 0.0121 (0.008 – 0.018) | 0.009 (0.006 – 0.014) | 0.82 (0.67-0.93) | 0.39 (0.04 – 0.73) | 0.56 (0.42-0.70) |
| 4 | 0.0158 (0.01 – 0.023) | 0.009 (0.007 – 0.014) | 0.66 (0.42-0.87) | 0.59 (0.31 – 0.83) | 0.61 (0.48-0.73) |
| 5 | 0.022 (0.015 – 0.031) | 0.008 (0.005 – 0.013) | 0.52 (0.21 – 0.81) | 0.72 (0.51 – 0.88) | 0.72 (0.59-0.84) |
| 6 | 0.029 (0.020 – 0.043 ) | 0.011 (0.006 – 0.018) | 0.41 (0.07 – 0.76) | 0.81 (0.64 – 0.92) | 0.72 (0.58-0.85) |

Table A2.4 Result for analyzes of the same data from Jolles et al. (2019), generated by the constant residual model. Note column B, which shows the estimated residual variance as a constant.

|  | A | B | C | D | E |
| --- | --- | --- | --- | --- | --- |
| Week | VARamg_t_ | VARresid_t_ | CORR_1,X_ | CORRe_t_,s | R_t_ |
| 1 | 0.014 (0.008 –0.02) | 0.013 (0.012 – 0.014) | NA | -0.40 (-0.62,-0.12) | 0.52 (0.39–0.63) |
| 2 | 0.011 (0.007 – 0.02) | 0.013 (0.012 – 0.014) | 0.94 (0.90–0.97) | -0.10 (-0.37 – 0.22) | 0.46 (0.35–0.57) |
| 3 | 0.012 (0.008 – 0.02) | 0.013 (0.012 – 0.014) | 0.77 (0.61–0.88) | 0.22 (-0.06 – 0.50) | 0.47 (0.37–0.57) |
| 4 | 0.015 (0.01 – 0.02) | 0.013 (0.012 – 0.014) | 0.53 (0.27–0.73) | 0.49 (0.23 – 0.70) | 0.53 (0.44–0.63) |
| 5 | 0.021 (0.014 – 0.03) | 0.013 (0.012 – 0.014) | 0.33 (0.04 – 0.59) | 0.67 (0.48 – 0.81) | 0.62 (0.52–0.71) |
| 6 | 0.030 (0.020 – 0.045) | 0.013 (0.012 – 0.014) | 0.18 (-0.12 – 0.46) | 0.77 (0.63 – 0.89) | 0.70 (0.60–0.78) |

2. Plots of diagnostics to assess whether the blups generated by each of the two models are normally distributed

These plots indicate that for the same data, the ‘blups’ generated by the constant residual model are more highly skewed than are the blups generated by the heterogenous residual model. Blups are the intercepts and slopes predicted by the model for each individual, expressed as deviations from the intercept and slope of the mean level model.

a. First, plots of the heterogenous residual model, using brms:

Intercept predictions Slope predictions


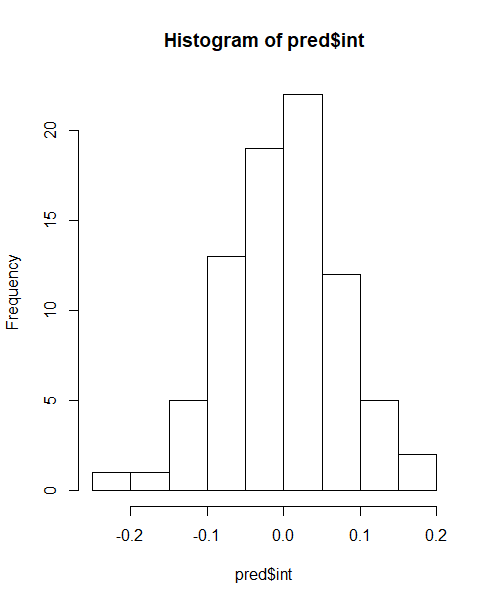

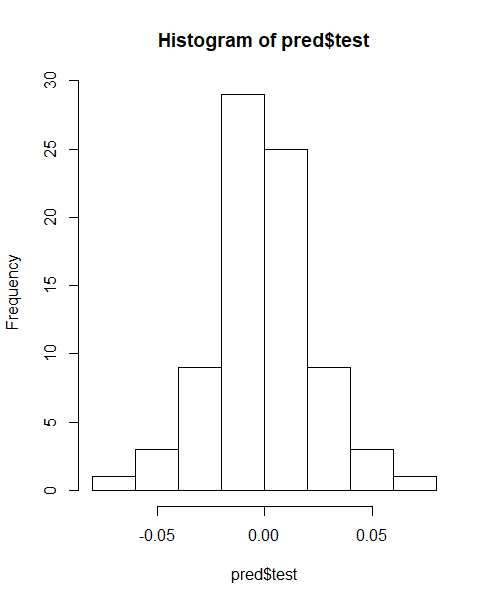


b. Second, plots from the constant residual model, using Lme4:

Intercept predictions Slope predictions


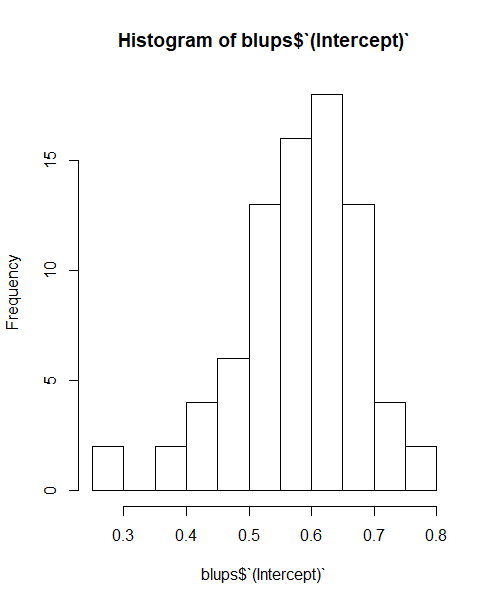

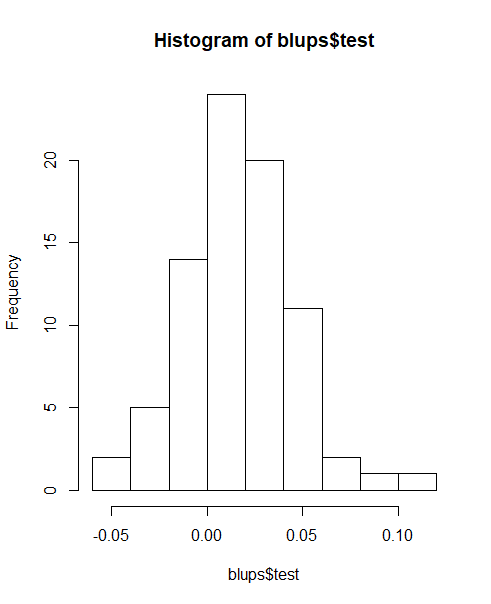


REFERENCES FOR APPENDIX S2

BIRO, P. A. & STAMPS, J. A. 2015. Using repeatability to study physiological and behavioural traits: ignore time-related change at your peril. *Animal Behaviour,* 105**,** 223-230.

BÜRKNER, P.-C. 2017. brms: An R Package for Bayesian Multilevel Models Using Stan. *Journal of Statistical Software* 80**,** 1-28.

JOLLES, J. W., BRIGGS, H. D., ARAYA-AJOY, Y. G. & BOOGERT, N. J. 2019. Personality, plasticity and predictability in sticklebacks: bold fish are less plastic and more predictable than shy fish. *Animal Behaviour,* 154**,** 193-202.

LASKOWSKI, K. L., BIERBACH, D., JOLLES, J. W., DORAN, C. & WOLF, M. 2022. The emergence and development of behavioral individuality in clonal fish. *Nature Communications,* 13.

**APPENDIX S3** Code in R for a worked example of the discrete time (character state) model, using data from Mitchell et al 2016

# Part A View data, plot data

# Part B Fit exploratory discrete time model using lme4

# Part C Fit discrete time model in brms

# Part D Calculations for VARamgt & Rt (note: CORRt1,t2 and its CIs are estimated directly)

# Part E Estimate CORRet,s using two random regressions

# Part F Extra bit: Comparison of results from the discrete time model with the results from the RR categorical time model

#################################################

#### PART A explore data, intitial analysis ####

#################################################

### set a working directory, read in data, check data ###

setwd("C:/Users/pbiro/Pete/Research/CONTINUING work/2020 temporal change w Judy")

ds<-read.csv("guppyiiv.csv")

summary(ds) # check how data is read-in

View(ds) # skim data, check

# View, transform response variable

hist(ds$ACT) # activity skewed, will affect residuals

ds$ln_ACT <- log(ds$ACT + 1) # transform

hist(ds$ln_ACT) # normal now

# Convert week and ID from factor to numeric to facilitate graphing/analysis

ds$Week <- as.numeric(ds$Week) # convert week to numeric (continuous) variable

ds$ID <- as.numeric(ds$ID) # convert raw id's to numeric variable

# Generate plots of data for each indvidual ID; annotations as in Suppl 1

z <- subset(ds, ID>50 & ID<63 )

library(ggplot2)

ggplot(z, aes(x = Week, y = ln_ACT, group = ID)) +

geom_point(size = 2) +

facet_wrap(. ~ ID)

theme_bw()

#################################################

#### PART B Prelminary discrete time model using lme4 ##

#################################################

library(lme4)

fit2<-lmer(ln_ACT ~ 0 + factor(Week) + (0 + factor(Week)|ID), data=ds, REML = TRUE)

summary(fit2) # removed Weight and ampm as predictors

######## quick plot of fitted individual trends from week to week ###################

library(broom)

r<-augment(fit2)

library(ggplot2)

ggplot(r, aes(x = factor.Week., y = ln_ACT, group = ID)) +

geom_line(aes(y = .fitted),

colour = 'red',

alpha = 0.9)

######## plot of fits against raw data, for subset of individuals ######

r$ID <- as.numeric(r$ID)

r2 <- subset(r, ID>50 & ID<63)

library(ggplot2)

ggplot(r2, aes(x = factor.Week., y = ln_ACT, group = ID)) + # same plot as before

geom_point(size = 2) +

geom_line(aes(y = .fitted), # adds lines for the fitted values

colour = 'red', alpha = 0.6) +

facet_wrap(. ~ ID)

#################################################

#### PART C Analysis using brms ###############

#################################################

# Now do analysis in brms to estimate CIs, and for residual modelling

library(brms)

# standardise (z transform) to facilitate analyses in brms

ds$ln_ACT <- (ds$ln_ACT - mean(ds$ln_ACT, na.rm=TRUE)) / sd(ds$ln_ACT, na.rm=TRUE)

# z-trans makes specifying priors easier, as these priors work for many kinds of models

lat<-brmsformula(ln_ACT ~ 0 + factor(Week) + (0 + factor(Week)|ID), sigma ~ 0 + factor(Week)) # also omit Weight and ampm here

fit1 <- brm(lat, data=ds,

prior = c(set_prior("normal(0,5)",class="b"),

set_prior("cauchy(0,2)",class="sd"),

set_prior("lkj(2)",class="cor")),

warmup = 500, iter = 2000, chains=4,

cores=4, control=list(adapt_delta=0.97))

summary(fit1) # provides model output

conditional_effects(fit1) # gives mean level trends plots; handy

plot(fit1) # posterior fits

mcmc_plot(fit1) # plots of params and intervals

## Now comes the rather complicated manipulations to get model predictions from output

### extract predicted values for each ID (aka the 'blups'), and fixed effects

id_blup <-ranef(fit1)$ID

fixed <-fixef(fit1)

View(id_blup) # view fitted blups to see structure,column names etc

### organise and tidy up predictions to make it useful for plotting ######

id <- data.frame(id_blup)

id$ID <- rep(row.names(id)[1:104])

View(id) # scroll way over to right to see new variable

# extract just the columns required

id<-subset(id,select=c("ID", "Estimate.factorWeek1","Estimate.factorWeek2","Estimate.factorWeek3"))

#convert the data frame "id" from 'wide' format to long format for plotting

a <- reshape(data=id, idvar="ID",

varying = c("Estimate.factorWeek1","Estimate.factorWeek2","Estimate.factorWeek3"),

direction="long")

a <- data.frame(a)

View(a) # check that it's worked!

# create new column for fixed effect prediction at each week

View(fixed) # first view structure of fixed effects

a$fixed[a$time=="factorWeek1"] <- fixed[1,1] # assign fixed effect=week 1 to new variable "fixed"

a$fixed[a$time=="factorWeek2"] <- fixed[2,1] # assign fixed (week 2)

a$fixed[a$time=="factorWeek3"] <- fixed[3,1] # assign fixed week 3

View(a) # check again its worked as planned

names(a)[3] <- "blup" # rename column 3 'Estimate' to make sense for reader

names(a)[4] <- "mean" # rename column 4 'fixed' to make sense for reader

a$time<-as.factor(a$time) # this line, and next, converts text values to 1-3

a$time<-as.numeric(a$time)

#plot predicted trend for each individual, as mean + blup

plot(NULL, xlim = c(1,3), ylim = c(-2.5,2.5),ylab="Activity",xlab="Time(weeks)")

for(i in 1:104){ # put range of IDs to plot here, 1-104 full range

x <- subset(a, ID == i) # filter data by ID into new data 'x'

lines(y = x$blup + x$mean, x= x$time, type = 'l') # plot each ID, as mean + blup, at each

}

# now add mean level trend line

a<-c(fixed[1,1],fixed[2,1],fixed[3,1]) # create column fixed effects (the y)

b<-c(1,2,3) # create column x values to go with y

lines(y = a , x = b, type = 'l',lwd = 3,col='red') # add mean trend

############################################################

#### PART D Calculate VARamgt and Rt ######################

############################################################

View(posterior_samples(fit1)) # view names of posteriors to use, but can refer to column no's too

# note the subtle column names where sd_ID__factorWeek1 has two underscores before Week! easy to miss!

library(coda)

VARWeek1 <- (posterior_samples(fit1)$"sd_ID__factorWeek1"^2) # get intercept sd and square

VARWeek2 <- (posterior_samples(fit1)$"sd_ID__factorWeek2"^2)

VARWeek3 <- (posterior_samples(fit1)$"sd_ID__factorWeek3"^2)

VARresid1 <- exp(posterior_samples(fit1)$"b_sigma_factorWeek1")^2 # get residual sd's, exp, then square

VARresid2 <- exp(posterior_samples(fit1)$"b_sigma_factorWeek2")^2 # the exp is because sigma is on log link

VARresid3 <- exp(posterior_samples(fit1)$"b_sigma_factorWeek3")^2

VARamg <- VARWeek3 # enter VARWeek value, 1-3

mean(VARamg);quantile(as.mcmc(VARamg),probs = c(0.025, 0.975)) # get mean and CI for VARamg

VARresid <- VARresid3 # enter resid week value

mean(VARresid);quantile(as.mcmc(VARresid),probs = c(0.025, 0.975)) # get mean and CI for VARamg

Rt <- VARWeek3/ (VARWeek3 + VARresid3) # R calc, just change week # and re-do for each wk

mean(Rt);quantile(as.mcmc(Rt),probs = c(0.025, 0.975)) # get mean and CI for Rt

#################################################

#### PART E Run separate RR's to estimate CORRets ####

#################################################

## here there are only two correlations of interest:

# CORRe1,s and CORRe2,s. therefore, we will run two analyses.

## start fresh and read in data, transform etc before running

setwd("C:/Users/pbiro/Pete/Research/CONTINUING work/2020 temporal change w Judy")

ds<-read.csv("guppyiiv.csv")

ds$ln_ACT <- log(ds$ACT + 1) # transform as before ...

ds$ln_ACT <- (ds$ln_ACT - mean(ds$ln_ACT, na.rm=TRUE)) / sd(ds$ln_ACT, na.rm=TRUE)

ds$Week <- as.numeric(ds$Week) # convert week to numeric (continuous) variable

ds$ID <- as.numeric(ds$ID) # convert raw id's to numeric variable

ds4<-subset(ds, Week < 3) # subset data to weeks 1-2

ds4$Week<-ds4$Week-1 # left centre for interval 1-2 contrast

ds5<-subset(ds, Week > 1) # subset weeks 2-3

ds5$Week<-ds5$Week-2 # left centre for interval 2-3

View(ds4)

View(ds5)

# now run model below twice, once using ds4 and then using ds5

# this model below treats time as continuous, permitting random slopes to be fitted

library(brms)

lat<-brmsformula(ln_ACT ~ 1 + Week + (1 + Week|ID), sigma ~ 0+ factor(Week)) # also omit Weight and ampm here

fit4 <- brm(lat, data = ds4,

prior = c(set_prior("normal(0,5)",class="b"),

set_prior("cauchy(0,2)",class="sd"),

set_prior("lkj(2)",class="cor")),

warmup = 500, iter = 1000, chains=4,

cores=4, control=list(adapt_delta=0.97))

summary(fit4)

# now re-run using ds5

##################################################################

#### PART F Comparison of results from the discrete time model with results from the RR categorical time model (see Supplement S4) ####

##################################################################

####################################

### fit RR categorical time model

ds$Week<- ds$Week-1 # new

lat<-brmsformula(ln_ACT ~ 1 + factor(Week) + (1 + Week|ID), sigma ~ 0+ factor(Week))

fitRR <- brm(lat, data=ds,

prior = c(set_prior("normal(0,5)",class="b"),

set_prior("cauchy(0,2)",class="sd"),

set_prior("lkj(2)",class="cor")),

warmup = 500, iter = 2000, chains=4,

cores=4, control=list(adapt_delta=0.97))

summary(fitRR)

####################################

### now fit discrete time model to compare

lat<-brmsformula(ln_ACT ~ 0 + factor(Week) + (0 + factor(Week)|ID), sigma ~ 0 + factor(Week))

fitBM <- brm(lat, data=ds,

prior = c(set_prior("normal(0,5)",class="b"),

set_prior("cauchy(0,2)",class="sd"),

set_prior("lkj(2)",class="cor")),

warmup = 500, iter = 2000, chains=4,

cores=4, control=list(adapt_delta=0.97))

summary(fitBM)

######################################

# asssess which model is better here

waic2 <- waic(fitBM) # discrete time model

waic1 <- waic(fitRR) # RR categorical time model

loo_compare( waic1, waic2)

**APPENDIX S4.** Using the discrete time model to analyze data collected using a burst design

The discrete time model is also referred to as a ‘character state’ model. It is often fit as a multivariate model, but we present a univariate equivalent here. In our discrete time model, time is specified as a factor for both the fixed effect and the random effect, and has the following form:

Y ~ 0 + factor(*time*) + (0 + factor(*time*) | ID). (10)

In this model, we do not fit an intercept (hence the zero in the equation) in either the fixed effects or in the random effects terms (in parentheses). As a result, the model fits a mean and among-subjects variance separately for each burst, rather than expressing predictions as deviations from an intercept. The time variable must be specified as a categorical predictor (factor) for this model to function in this way, in contrast to random regression, where time is usually a continuous predictor for the fixed effect. Thus, for a model in which data was collected in three bursts (as in our worked example, see below), there would be three fixed effect parameters estimated for the population level response, three among subject variances, and three pairwise covariances at the individual level. Consequently, VARamg_t_ is estimated directly for each burst and therefore requires no further calculations (unlike RR). CORR_t1,t2_ is also estimated directly by the model, based on the estimates of each subject's mean values for all possible pairs of bursts.

Finally, we add a residual side model and fit a separate residual sd for each level of time as:

sigma ~ 0 + factor(*time*) (11)

Given that the values of VARamg_t_ and VARresid_t_ for each burst are estimated directly in this model, estimating R_t_ for each burst would rely on Eqn. 9 in Appendix S2, using the code indicated in Appendix S3C to estimate the CIs for the estimate of R_t_ for each burst.

One disadvantage of the discrete time model is that it does not allow us to compute CORRe_t_,s the correlation between each subject's mean value in one burst, and the change in its mean value from that burst to the next burst (its slope). We suggest using the random regression model described in the previous section to compute CORRe_t_,s for burst data: this would involve choosing pairs of bursts for analysis, and then treating the time between those bursts as a continuous variable. However, it is worth noting that this is not a parsimonious approach, since it requires separate analyzes for each pair of bursts that one chooses to consider. If there were several or more bursts, then fitting a random regression model with polynomial terms might be appropriate and useful (see Discussion in the text).

A. A worked example using data from Mitchell et al. (2016) (see text).

We begin by plotting the data for all the subjects to inspect the temporal trends across the three bursts of sampling (for code, see Appendix S3). Even after ln (x + 1) transformation, these plots suggested that the reaction norms of most of the subjects were nonlinear over the three burst periods, and the shapes of the reaction norms varied considerably among individuals, ranging from linear to strongly non-linear (Figure A4.1).


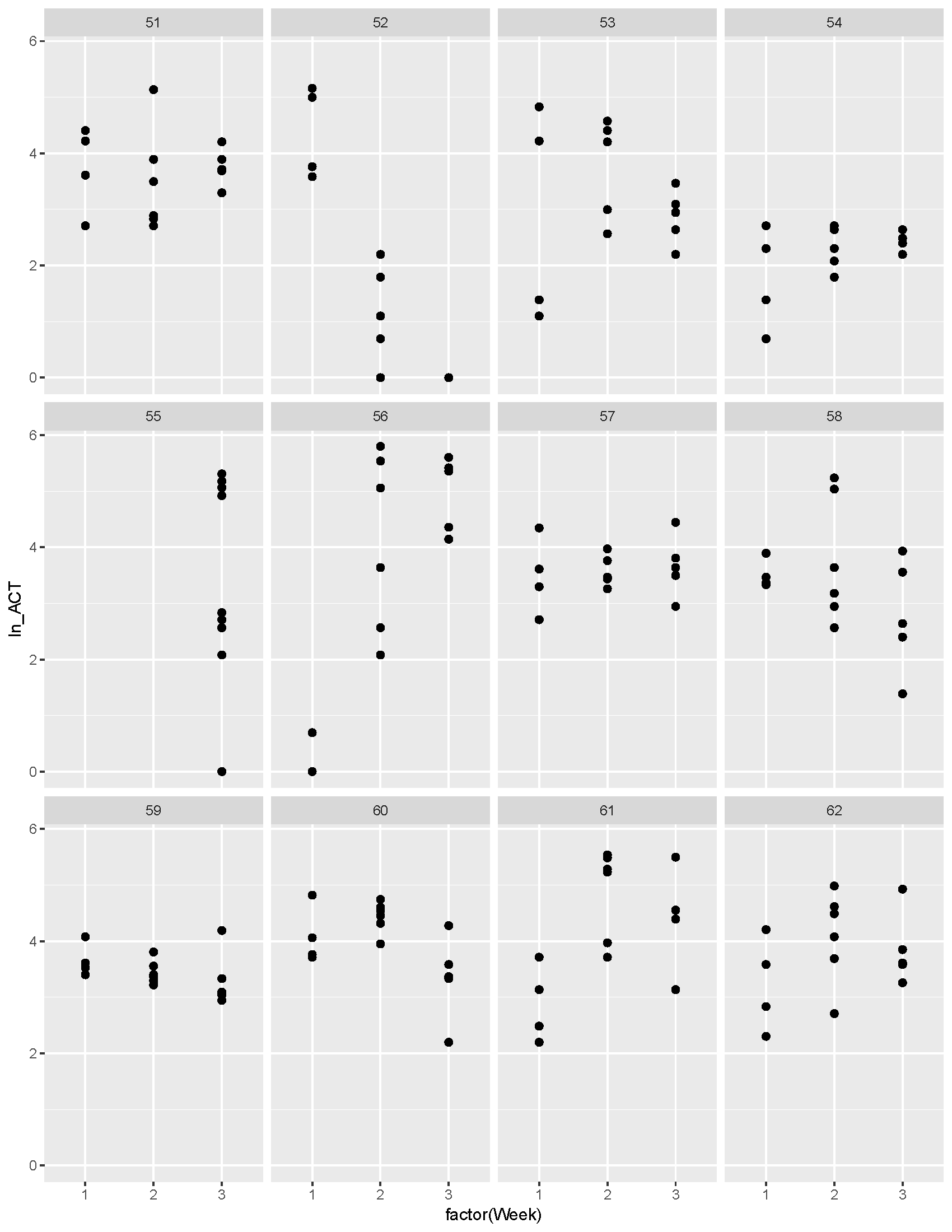


Figure A4.1. Activity rates of individual male guppies using data from Mitchell et al. (2016). Shown are the ln(activity +1) transformed activity rates across three weekly bursts of data collection for an arbitrary subset (ID numbers 51 – 62) of the subjects in the study.

In an exploratory analysis using the program lme4 (which assumes residual variation is constant), we fit the above model and obtain the results indicated in Table A4.1.

Table A4.1. Results for data in Mitchell et. al. (2016): exploratory analysis for a discrete time model

(using lme4 in R)

Random effects:

Groups Name Variance Std.Dev. Corr

ID factor(Week)1 0.7981 0.8934

factor(Week)2 0.6725 0.8200 0.46

factor(Week)3 0.7942 0.8912 0.28 0.73

Residual 0.6503 0.8064

Number of obs: 1477, groups: ID, 104

Fixed effects:

Estimate Std. Error t value

factor(Week)1 2.99584 0.10052 29.80

factor(Week)2 3.35845 0.08830 38.04

factor(Week)3 3.03616 0.09559 31.76

In Table A4.1, under Random effects, Variance, we see the estimate of VARamg_t_ for each of the three weeks. These results suggest that VARamg_t_ might have been slightly lower in week 2 than in either week 1 or 3. The “Corr” values provide an estimate of CORR_t1,t2_ in matrix form: the correlation between the subjects' mean values at one burst and their mean values at another burst. All three pairwise correlations are positive, suggesting that some level of rank-order was maintained among the subjects, especially between bursts 2 and 3 (r = 0.73). Next, we ask whether the exploratory model does a reasonable job of predicting the temporal reaction norms for the subjects. As is shown in Figure A4.2, this is the case. In addition, the model not only confirmed our initial impression that the individual trendlines were non-linear over the course of the three week study period, but also indicated that the shapes of the reaction norms varied considerably among the subjects. In addition, many of the subjects seemed to have higher mean values at week 2 than at weeks 1 or 2, which is evident in the fixed effect estimates for week 1, 2 and 3 in Table A4.1.


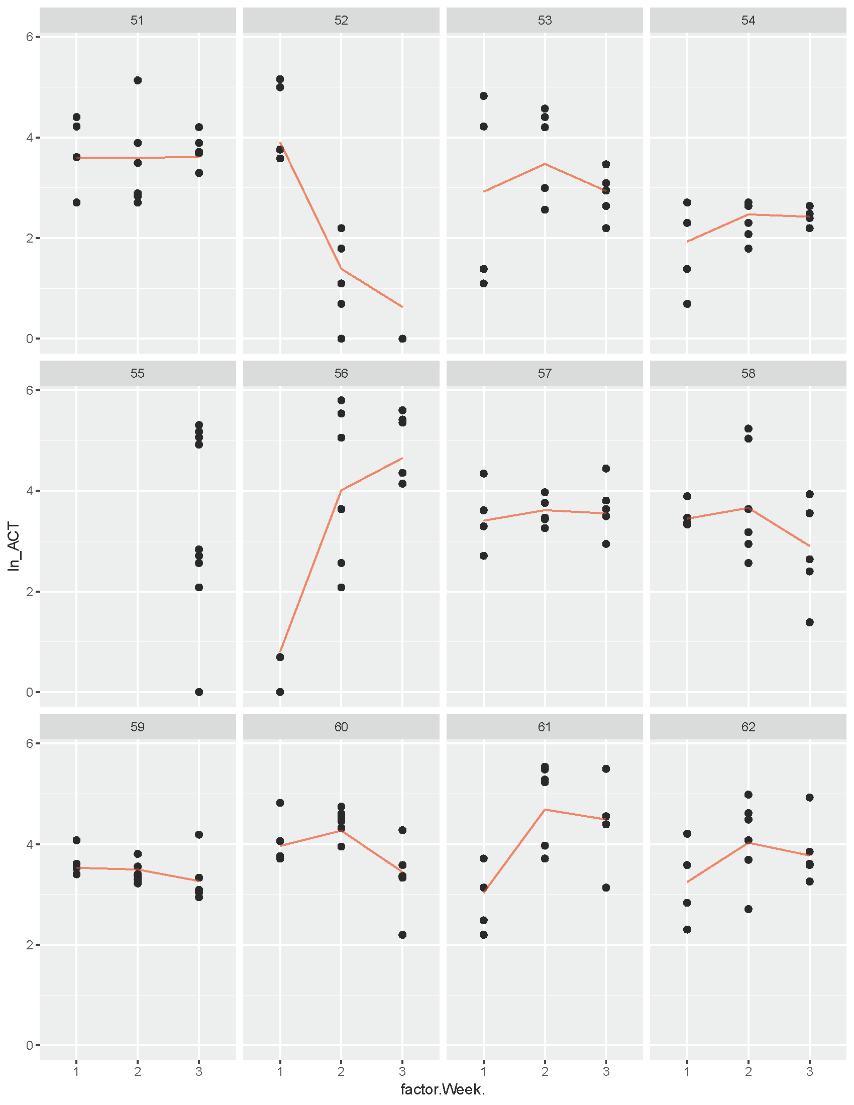


Figure A4.2. Replication of the individual plots in Figure A4.1, but now with the trendlines indicated by the exploratory model added at each burst and connected by lines across bursts (in red) to indicate the temporal reaction norms of each subject.

Based on the results of the exploratory model, we next fit the same data using the program brms. As was the case for the random regression model, brms allows us to compute CIs for the variables of interest and allows residual variation to vary over time (code in Appendix S3). Plots of the fitted reaction norms and the mean level trend suggest a) a slight increase in activity in week 2 compared to weeks 1 and 3, b) highly variable and in some cases highly non-linear trends for some individuals, c) substantial crossing over of reaction norms between weeks 1 and 2, and d) lower levels of crossing over between weeks 2 and 3 (Fig. A4.3). However, with so many individuals, the graph is difficult to interpret without our supporting statistics.


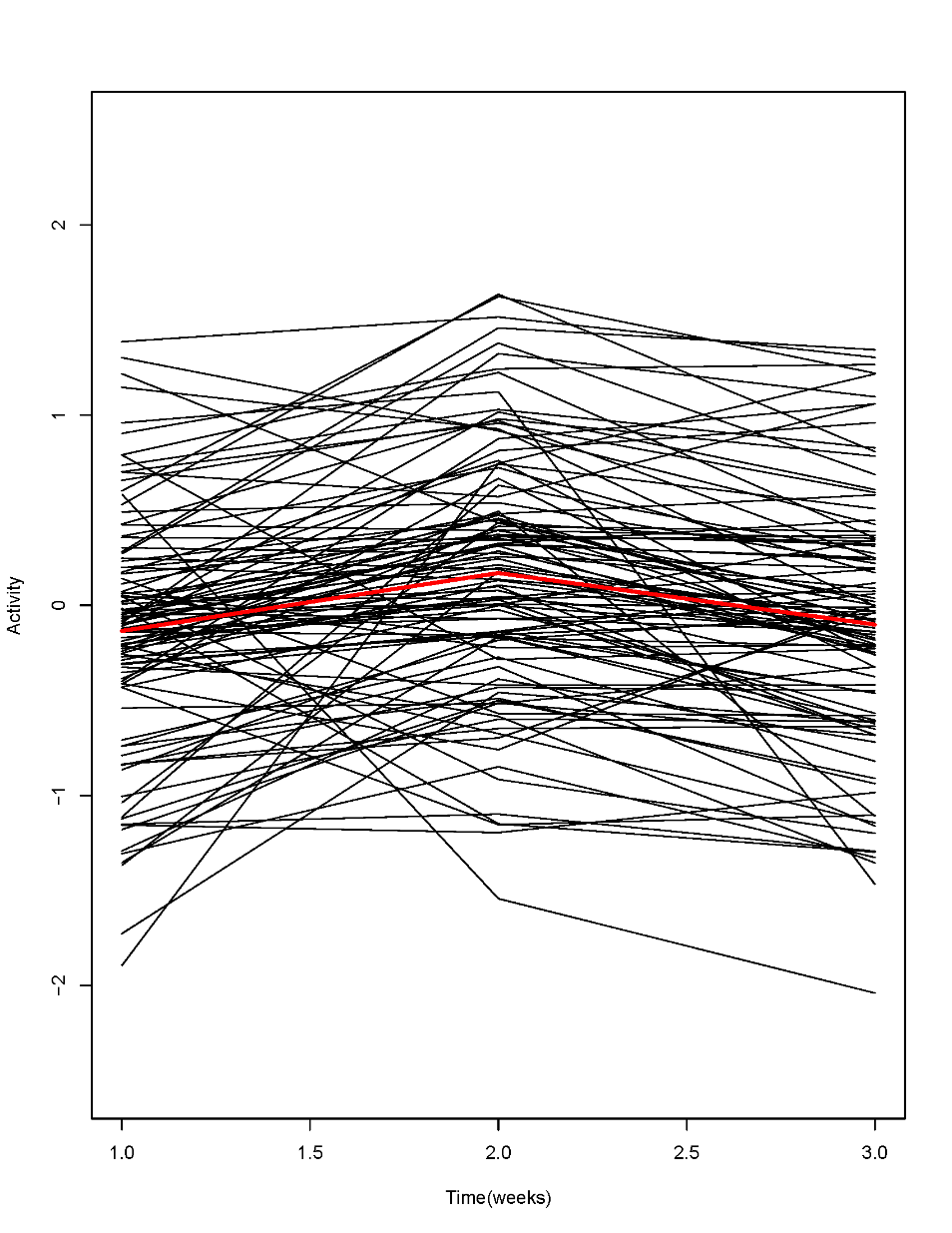


Figure A4.3 Fitted temporal trendlines of guppy activity rates (from three bursts of sampling, generated from a discrete time (‘character state’) type model which permits among subjects' variance and residual variation to vary over time. Shown are 104 individuals, with the mean level trend shown in red. Activity is expressed in units of SD following transformation (see Appendix S3A).

The results of the analyzes of the data in Mitchell et al. (2016) indicate that VARamg_t_ was similar in weeks 1, 2 and 3, with broadly overlapping CIs for this variable across the three-week period (Table A4.2, column A). However, VARresid_t_ did change over time; the 95% CIs for this variable indicated that VARresid_t_ was significantly lower in week 2 than it was in either weeks 1 or 3 (Table A4.2, column B). In addition, rank-order consistency varied over time: CORR_t1,t2_ was somewhat maintained between weeks 1 and 2 (r = 0.44), strongly maintained over weeks 2 and 3 (r = 0.69), with a weak tendency for maintenance over the entire study (r = 0.26); Table A4.2, Columns B and C).

Table A4.2. Estimates for VARamg_t_, VARresid_t_, CORR_t1,t2_, and R_t_ presented for different time points, based on discrete time and random regression analyzes of data from Mitchell et al. (2016). Mean and CIs are indicated for each variable.

|  | A | B | C | D | E |
| --- | --- | --- | --- | --- | --- |
| Week (X) | VARamg_t_ | VARresid_t_ | CORR_1,X_ | CORR_2,3_ | R_t_ |
| 1 | 0.56 (0.39 - 0.80) | 0.55 (0.46 – 0.64) | NA |  | 0.50 (0.40- 0.61) |
| 2 | 0.50 (0.37 - 0.67) | 0.36 (0.31 – 0.41) | 0.42 (0.22- 0.60) |  | 0.58 (0.50 - 0.66) |
| 3 | 0.56 (0.39 – 0.76) | 0.53 (0.47 – 0.61) | 0.26 (0.04 - 0.47) | 0.69 (0.53 - 0.81) | 0.51 (0.41 – 0.60) |

Estimation of CORRe_t_,s using two separate RR analyzes (see Appendix S4.E) revealed that this variable was strongly negative from week 1 to week 2, indicating convergence during that period (r = -0.52, CIs: -0.69 – -0.33). In contrast, CORRe_t_,s from week 2 to 3 was weakly negative, with an upper CI that nearly overlapped zero, suggesting that modest, if any, convergence occurred during this period (r = -0.29, CIs: -0.50 – -0.05).

B. Comparison of two options for analysis of temporal changes in individual differences when data is collected using a burst design

Here we use the data on guppy activity from Mitchell et al. (2016) to illustrate the ‘pros’ and ‘cons’ of two different statistical models that can be used to capture temporal trends for data collected in bursts.

Figure A4.4, left panel: predictions from a 'RR categorical time' model, a random regression model which assumes time is a factor at the mean level, but continuous on the random effects, using the following R syntax:

Activity ~ 1 + factor(time) + (1 + time | ID).

For examples of studies which have used this type of model to analyze temporal changes in individual (and/or genotype) reaction norms, see Dingemanse et al., 2012 and Class et al., 2019.

Figure A4.4, right panel: predictions from the 'discrete time' model described in the text and above, in Appendix S4A. In this model, fixed and random effects are fit for each level of time, using this syntax:

Activity ~ 0 + factor(time) + (0 + factor(time)|ID).

Figure A4.4. Comparison of the temporal reaction norms for the subjects in Mitchell et al. (2016), based on the RR categorical time model (left panel) and the discrete time model (right panel)

RR categorical time model Discrete time model


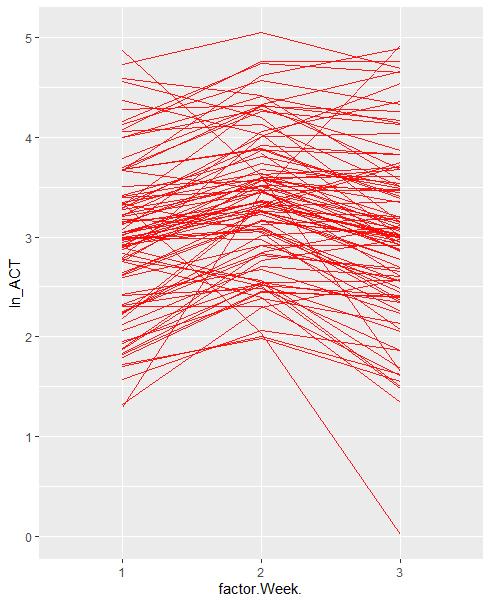

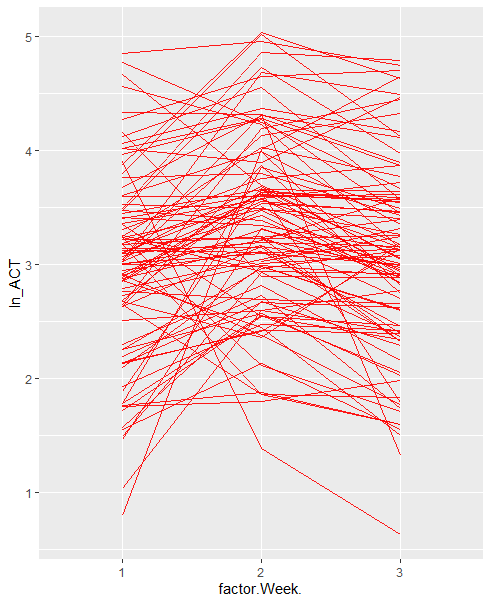


Note how the RR categorical time model constrains the shapes of the individual's reaction norms to be similar to the shape of the mean trend at the population level, which may be an limitation of this approach. By contrast, the more flexible discrete time model captures variation among the subjects in the shapes of their reaction norms. In addition, the discrete time model provides a much better fit to this data set than does the random regression model (delta WAIC = 59.2, see code in Appendix S3). A potential downside of the discrete time model is that it requires more parameters to fit, particularly if there are more than a few levels of time, and it requires repeated measures at each level of time.

REFERENCES FOR APPENDIX S4

CLASS, B., BROMMER, J. E. & VAN OERS, K. 2019. Exploratory behavior undergoes genotype-age interactions in a wild bird. *Ecology and Evolution,* 9**,** 8987-8994.

DINGEMANSE, N. J., BARBER, I., WRIGHT, J. & BROMMER, J. E. 2012. Quantitative genetics of behavioural reaction norms: genetic correlations between personality and behavioural plasticity vary across stickleback populations. *Journal of Evolutionary Biology,* 25**,** 485-496.

MITCHELL, D. J., FANSON, B. G., BECKMANN, C. & BIRO, P. A. 2016. Towards powerful experimental and statistical approaches to study intraindividual variability in labile traits. *Royal Society Open Science,* 3.
